# The Epidemiology of Plant Virus Disease: Towards a New Synthesis

**DOI:** 10.3390/plants9121768

**Published:** 2020-12-14

**Authors:** Michael J. Jeger

**Affiliations:** Department of Life Sciences, Imperial College London, Silwood Park, Ascot SL5 7PY, UK; m.jeger@imperial.ac.uk

**Keywords:** epidemiology, ecology and evolution of plant viruses, mathematical modelling, transmission, vector population dynamics, behaviour and preferences, coinfection

## Abstract

Epidemiology is the science of how disease develops in populations, with applications in human, animal and plant diseases. For plant diseases, epidemiology has developed as a quantitative science with the aims of describing, understanding and predicting epidemics, and intervening to mitigate their consequences in plant populations. Although the central focus of epidemiology is at the population level, it is often necessary to recognise the system hierarchies present by scaling down to the individual plant/cellular level and scaling up to the community/landscape level. This is particularly important for diseases caused by plant viruses, which in most cases are transmitted by arthropod vectors. This leads to range of virus-plant, virus-vector and vector-plant interactions giving a distinctive character to plant virus epidemiology (whilst recognising that some fungal, oomycete and bacterial pathogens are also vector-borne). These interactions have epidemiological, ecological and evolutionary consequences with implications for agronomic practices, pest and disease management, host resistance deployment, and the health of wild plant communities. Over the last two decades, there have been attempts to bring together these differing standpoints into a new synthesis, although this is more apparent for evolutionary and ecological approaches, perhaps reflecting the greater emphasis on shorter often annual time scales in epidemiological studies. It is argued here that incorporating an epidemiological perspective, specifically quantitative, into this developing synthesis will lead to new directions in plant virus research and disease management. This synthesis can serve to further consolidate and transform epidemiology as a key element in plant virus research.

## 1. Introduction

Epidemiology is the study of how disease develops in populations [1], in the context of plant disease epidemics, the change in disease intensity in a host population over time and space. The term population is used here to denote a group of individuals bounded by both spatial and temporal parameters with the potential for genetic exchange among individuals. This definition emphasises the need for quantification, but also that there is a need for theory to underlie observational and experimental studies in epidemiology [2]. A traditional view of epidemiology is that, progressively, it has firstly the aim of description, how best to describe epidemics as a spatiotemporal pattern. Secondly, having described the epidemic, to aim to understand the dynamic changes in the pattern observed. Thirdly, and based on the understanding gained, to predict the future changes. Finally, to decide when and how to intervene to prevent or mitigate the consequences of epidemics, such as crop loss or biodiversity loss, although in cases of severe outbreaks there is often the imperative to intervene without necessarily having a full understanding of the underlying epidemiology. In these cases, a balance needs to be struck between prediction based on interpretation of observational data, made easier nowadays by new data mining and computational techniques, and the need to generate a mechanistic understanding—the implication being that empirical prediction of necessity precedes explanation.

**Figure 1:**
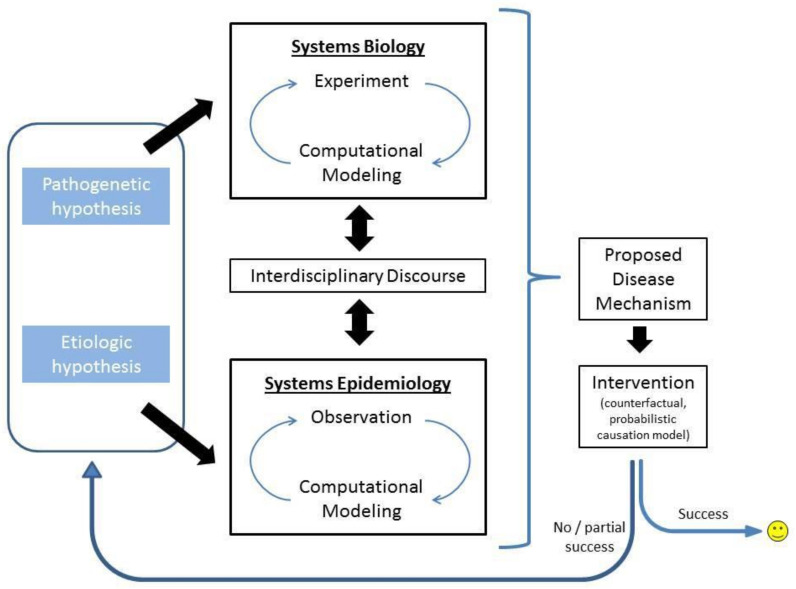
Conceptual view of hierarchies in epidemiology, Damman et al. 2014 [3], (Figure 1). The “systems biology” concerns experimental research and modelling of pathogenesis at the cellular level; “systems epidemiology” refers to observational research and modelling of disease aetiology at the population level. For both levels there is overlap with the organismal level. Both levels of integration are necessary for the success of control interventions.

This sequence raises questions concerning the epidemic as a hierarchical system with nested levels of integration. For example, if the aim is to understand the rate of change in disease at the population level, it would be necessary to describe processes at the individual plant level (and in some cases down to the cellular level); equally, if an understanding of disease at a higher level, such as in natural plant communities or over landscapes, then at the very least a description of processes at the population levels is required. This leads naturally to thinking in terms of systems epidemiology [3] and the need to integrate across different levels of integration (Figure 1). Although directed at medical epidemiology, the principles are equally applicable in plant disease epidemiology. Systems epidemiology provides a gene-to-landscape vision for understanding plant-microbe interactions. A counter view is that in a system composed of hierarchical levels, there are emergent properties at the higher level that are not predictable even where there is a full understanding of the processes and interactions at the lower level. In this review, the emphasis will be placed on the population level, with descriptions at the organismal/cellular level, and showing how such description is appropriate for analysis at the community/landscape level. This is not to say that all epidemiological studies need to follow this approach, which in some cases may not be realisable, but they should at least evaluate at the outset the levels of integration that need to be considered in a research plan.

**Figure 2:**
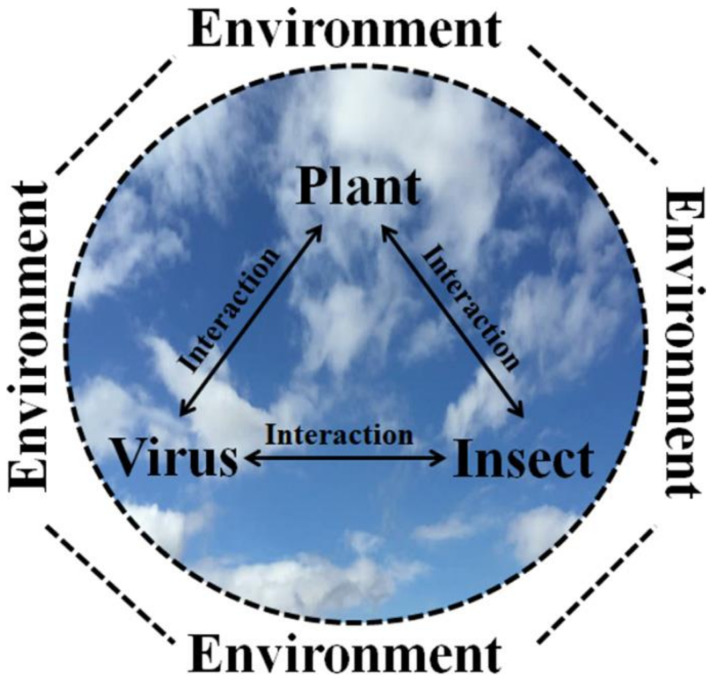
Extending the disease triangle concept to vector-borne diseases, Islam et al. 2020 [5], (graphical abstract). The plant, the virus, and the host are represented as the corners of the triangle, within a surrounding abiotic environment. A more revealing representation would place the three 2-way interactions at the corners and to consider the broader environment as both biotic and abiotic.

A concept with a long history in plant pathology is the disease triangle, stressing the combination of a susceptible host, a virulent pathogen and a conducive abiotic environment as being essential for disease. Such a concept has it pedagogical uses but is often used in such a static way that it is questionable whether it provides any real insight into the processes underlying plant disease. As has been pointed out [4], there needs to be recognition that each of these three elements, host, pathogen and environment, can indirectly affect each other in a dynamic way that can modify the disease outcome. Further, most plant viruses are transmitted by vectors and this adds a level of complexity to the disease triangle as shown in Figure 2 [5]. An alternative to this representation would be to replace the three triangle corners by: (a) the virus-plant interaction, (b) the plant-vector interaction, and (c) the vector-virus interaction. These interactions represent Walter Allen’s “inseparable ecological trinity” of virus, vector and host plant [6] and can better represent plant virus disease. A similar view was expressed on the complexity of the “three cornered” interaction of virus, vector and host in relation to prediction of plant virus disease epidemics [7]. Although other plant pathogens, including fungi, phytoplasmas, bacteria, oomycetes and nematodes, may be associated with vectors, these interactions give a distinctive character to plant virus epidemiology, with consequences for agronomic practices, pest and disease management, host resistance deployment, and disease in wild plant communities including weeds. It is important to recognise that the biotic as much as the abiotic environment is an equal consideration for vector-borne diseases because of the tripartite and tritrophic interactions between vectors and related or unrelated biota. This consideration applies particularly to arthropod vectors as described in later sections of this review.

## 2. Plant Virus Epidemiology, Ecology and Evolution

There have been many overviews of the place of plant virus epidemiology in plant virology research over the last two decades with its distinctive character brought together in an integrated way. It should be borne in mind that most are selective and not representative of the full range of plant viruses, which currently covers some 1516 species across 26 families (10th Report of the International Committee on Taxonomy of Viruses (ICTV2018b.v1)). In this review, for example, some 40 species only are covered in the research publications cited. A conventional view of plant virus disease epidemiology would emphasise how disease spreads in time and space, the role of vectors and the wider ecosystem, the interactions between viruses, vectors, host plants and the environment, and how knowledge of these interacting factors can improve disease control in different cropping systems [8,9,10]. However, it is also in the writer’s experience that in many national and international symposia and fora on plant virus epidemiology, papers are often presented on topics such as high-resolution virus characterisation techniques or the fine structure of vector anatomy without the relevance for disease epidemiology, as defined above, being shown. This, perhaps, would show a distinction from presentations at similar fora for fungal disease epidemiology.

Technological developments in support of research in plant virus epidemiology take many forms Many examples of recent developments and their application for a range of case studies have been reviewed [11], including: (a) remote sensing at scales ranging from crop in-field monitoring using drones to continents using satellite technology; (b) information and decision-support systems to assist in the prediction of epidemics; (c) analytical methods for temporal and spatial spread within crops for a better understanding of the consequences of disease control interventions; (d) advances in molecular epidemiology made possible by advances in virus detection and analysis of genetic variation; and, importantly, (e) given the global issues relating to food security, climate change and biodiversity, the need to develop and rapidly adopt new technological advances [11]. Similar advances have been made in the application of mathematical models for an understanding of epidemics and for disease control [12]. Areas covered include the better use of knowledge of environmental factors, spatial and temporal analysis, the consequences of disease control interventions, production systems and plant community dynamics, and with special attention given to the importance of considering vector life cycles, behaviour, population dynamics and transmission characteristics in mathematical models [13]. However, despite the significant advances made in linking mathematical models with biological realism and data, many gaps remain, providing opportunities for future research and the impact on disease management practices remains questionable. There are now real opportunities for further synthesis by a greater consolidation and extension of ecological and evolutionary insights into epidemiological analysis of the causes and consequences of plant virus disease epidemics; in particular, by recognising the shorter-term (ecological) and longer-term (evolutionary) consequences of disease epidemics.

### 2.1. Epidemiology and Evolution

There has long been recognition that a closer integration of plant disease epidemiology and evolutionary biology would add substantially to both our understanding of epidemic dynamics and the prospects for disease management. This certainly applies across all pathogen taxa, including plant viruses, in terms of the synthesis of epidemiology and population genetics [14]. For plant viruses, an approach based on the concept of evolutionary epidemiology has been proposed [15], arguing that some epidemiological components can only be appreciated by incorporating ecological and evolutionary ideas into more traditional epidemiology. In a similar vein, the emergence and evolution of plant viruses is intimately related to disease epidemiology through epidemic dynamics, effects mediated through transmission, and virus manipulation of the vector [16].

Tracing the origin and evolutionary pathways of plant viruses during crop domestication has proved difficult [17]. To put this into context, flowering plants emerged during the Cretaceous some 130 million years ago, together with insect pollinators, although molecular dating suggests possibly earlier [18]. The common ancestor of extant families in the Aphididae arose in the late Cretaceous [19]. The history of domestication of well-known crops in the Poaceae dates back some 10,000 years but information on other crops is more limited [20]. The oldest plant virus known was dated from 1000 years ago, from maize cobs in an archaeological site [21], a member of the chrysoviruses, which are transmitted through seeds. This context makes interpretation of often rapid adaptive changes in relation to agronomic or ecological factors difficult, even where the molecular bases for such change can be determined [22]. The high rates of population change in RNA plant viruses mean that there are valuable insights into their later evolution during the period of crop improvement and intensification that can be used to look at how humans have influenced the development of disease. Good examples of these are the growing series of papers on potato viruses using ‘historic’ and land race isolates and comparing these with current isolates from around the world [23,24,25].

The evolution of plant viruses with beneficial rather than harmful effects in crop plants [26,27] raises further questions that need addressing. For example, integration of viral genome sequences into the host genome may confer beneficial effects such as enhanced virus resistance [28] that may be expressed in field populations. On the other hand, in wild populations of *Arabidopsis thaliana*, it has been shown that cucumber mosaic virus (CMV) is a highly virulent pathogen, but with resistance and tolerance to the virus having evolved from other adaptive traits [29]. The evolution of both parasitic and mutualistic plant viruses has been modelled [30] and showed that both trajectories—from parasitic to mutualistic, and from mutualistic to parasitic—were possible depending on virulence and transmission characteristics. In the shorter-term, parasitic and mutualistic strains can co-exist and in the longer-term mutualism can dominate. An outstanding question is whether epidemiological analysis as currently applied is appropriate for the population biology of mutualistic plant viruses [31], as would be the case for other mutualists such as rhizobia and mycorrhiza.

Even in the shorter-term, the deployment of plant varieties with high levels of qualitative resistance to plant pathogens may lead to the development of pathogen genotypes which render the resistance ineffective. A model was developed in which epidemiological and evolutionary processes influence emergence of plant virus genotypes that infect previously resistant host genotypes [32]. A global sensitivity analysis of the model shown population genetic parameters, including number of mutations and fitness of mutant genotypes had a major effect on the probability of resistance becoming ineffective. However epidemiological parameters, including the infection/contact rate and the host latent period, also affected the variation found, stressing that integrating evolutionary and epidemiological perspectives and approaches are essential in improving disease management through host resistance deployment.

### 2.2. Epidemiology and Ecology

Additionally, ss well as the need to include an evolutionary perspective in plant virus epidemiology, there is a need and indeed a trend to bring in an ecological perspective on the role of plant viruses in both managed and unmanaged ecosystems [33]. This added perspective has the potential to enrich plant ecology theory and research; reciprocally, plant virus epidemiology can benefit from developments both conceptual and practical in the ecological sciences. One area that illustrates this is landscape epidemiology [34]. In this approach (which can be applied to all plant pathogen taxa), landscape features of heterogeneity and fragmentation, defined rigorously in terms of composition, structure, and other properties, affect pathogen prevalence, pathogen dispersal and spread of disease, and pathogen emergence. Virus examples used to illustrate the approach include plum pox virus, barley/cereal yellow dwarf diseases, CMV, whitefly transmitted viruses, and rice yellow mottle virus.

Recently, the various ways in which virus life history traits and ecology affect population dynamics and evolutionary potential have been reviewed [35], considering population genetic traits of mutation and recombination, bottlenecks, and selection, together with host range and transmission mode. Further, the authors suggest that disease management would be improved by a better understanding of the “links between virus life history, population dynamics, and evolution”, although specific examples of how such improvement has/could be achieved are not given. The value of the review is that comparisons are made with other plant pathogens, but also with vector-borne animal, including human, viruses. There is a danger in this approach that theory developed for other organisms may lead to generalisations that are not always appropriate for plant viruses.

### 2.3. Further Synthesis

A full picture of the relationship between virus origin and evolution and host range must include ecological and epidemiological factors and these present new challenges for plant virus research. This can be seen from three perspectives: ecosystem ecology, viral community ecology, and epidemiology the host-virus-vector interaction [31]; a view considered an “exciting new field of research” for virus emergence [36]. In both cases [31,36] there is an emphasis on the impact on wild-plant communities and the role played by refugia. A further development linking ecological and epidemiological approaches has been described as phylodynamics [37], in which “Phylodynamics analyses have mostly focused on: (a) understanding when the current genetic diversity of plant virus populations was originated; (b) exploring how host defences and current strategies for disease control affect virus evolution and epidemiology; (c) identifying the origin and dispersion patterns of plant viruses at different landscape scales; and (d) analyzing the ecological factors shaping the evolution and epidemiology of plant viruses.”

In the synthesis of epidemiology, evolution and ecology, there should be a greater recognition that domesticated crop plants, even in extensive monocultures and other restricted cropping systems with less biodiversity also have an ecology, not just the wild or relatively unmanaged plant communities which are often used as exemplars for an ecological approach [20,27,38]. For many viruses ‘known host range’ is listed as several tobaccos, two or three *Chenopodium* spp. and if lucky a couple of commercially important crops, i.e., neither the experimental host range nor is ‘real world’ knowledge of virus distribution available. This point was taken up in relation to the “burden of proof” [39].

Finally, there is much to be gained by comparing the evolution, ecology and epidemiology of plant viruses [40] with the work done with a broader range of host–parasite interactions [41,42].

## 3. Epidemiology and Disease Control

Epidemiological analysis and mathematical models have long been used in support of forecasting and control of pathogens of human, animal and plants. However, despite some similarities in the modelling techniques, approaches to forecasting and control have developed independently and diverged [43], which is not surprising given the overwhelming dominance of modelling studies on human diseases. The view that epidemiological analysis, and in some cases modelling, can lead to improved disease management remains a testable proposition not a demonstrable fact [44], although this can be substantiated in many cases. In this section some examples are given which demonstrate the value of quantitative methods in epidemiology and disease control.

### 3.1. Epidemiological Analysis

The previous section highlighted some of the key overviews that provide some synthesis of evolutionary, ecological and epidemiological approaches in understanding plant virus epidemics. It is not clear how much this synthesis has contributed to an improved management of virus diseases, especially those of economic crops. Hence it is first necessary to show how epidemiological analysis has contributed to describing, understanding and predicting plant virus epidemics, and how the insights gained have contributed to disease management, whether in terms of cropping practices, host resistance deployment or forecasting based on weather or other environmental factors. The examples given are intended to be informative but not necessarily representative of all studies.

The first point to be made is that complete knowledge of a pathogenic agent is not necessarily a pre-requisite for disease control. For example, Bahia bark scaling of citrus is a disease of unknown aetiology, although analysis of its distribution suggests local dispersal by an insect vector. Even though of unknown aetiology, cost-effective methods of control can be devised based on this analysis [45]. Conversely, in some cases, diseases previously thought to be associated with a virus have subsequently been shown to be caused by an insect-borne bacterium, for example Pierce’s disease of grapevine caused by *Xylella fastidiosa* [46]—a “paradigm” that, it is claimed, held back and misdirected research for many years.

Secondly, there are many examples of plant viruses which are well-characterised and have been long studied, such as plum pox disease, where epidemiological analysis has proved fruitful. Plum pox virus (strain D) was first discovered in Ontario, Canada, in 2000. An eradication programme was put in place based upon constant surveillance combined with stringent removal of infected plants and block plantings. Despite these protocols, eradication had not been achieved at the time of the programme’s termination in 2011 [47]. The reasons suggested for this failure were that viruliferous aphids transmitted the virus most commonly some 43 m distance from previous infections and that there was a long tail of infections stretching out at least one order of magnitude further. From these analyses, a range of removal distances was proposed based on a risk-based methodology, but it is unclear what follow-up action has been taken. In Europe, strains of virus have been present for much longer, over the last century. Plum pox virus (strain M) disease was monitored for up to 10 years in peach orchards in France [48]. Symptomatic trees were removed each year and disease incidence in general remained low in most orchards. Despite the continuing rogueing effort, symptomatic trees were detected over this period. Analysis of an extensive set of locational, biotic and abiotic factors suggested that new infections arose from outside inoculum from neighbouring diseased orchards or because symptomatic trees were missed, or latently infected trees were not detected during visual inspection.

Long-term (1980–2011) spatial data on pine wilt disease (PWD), caused by the pathogenic nematode *Bursaphelenchus xylophilus*, and vector abundance of *Monochamus* spp. beetles, in the northern Japanese mainland, was analysed [49]. The data were fitted to a multiple state occupancy model in which categorical states of infection were specified (low, medium, high) and allowing for demographic stochasticity and observation error. Extensive meteorological and land-use data were included in the analysis. Parameters of the model were estimated using the Markov chain Monte Carlo method (MCMC). Results showed there was a positive density dependence (Allee effect) in vector abundance and a weaker climatic (degree day accumulation) effect restricting invasion of the beetle in northern regions. It was considered that removal by logging of infected pines would strengthen the Allee effect and slow down the spread of PWD.

The MCMV approach was applied recently to data on the spread of banana bunchy top virus disease (2014–2018) in a banana plantation in New South Wales, Australia [50]. Parameter estimates were obtained for a stochastic spatial susceptible-infected-susceptible model of disease spread and suggested seasonality in all estimates, influenced by inspection accuracy, temperature and aphid activity. The results of the analysis could be used for improving surveillance and forecasting and potentially be useful in policy-level decisions on managing the disease.

### 3.2. The Basic Reproduction Number

The basic reproduction number *R*_0_ and its extensions [51] have become key theoretical concepts in infectious disease modelling. The *R*_0_ value gives the number of infected ‘entities’ that would arise from the introduction of a single infectious entity into a susceptible host population during that entity’s period of infectiousness and provides a means of guiding control strategies, as has been shown in many studies in animal and human health, including the current COVID-19 pandemic. Besides its conceptual value, methods have been developed for estimating *R*_0_ during the early stages of a disease outbreak when information on the initial growth rate of an epidemic is available [52]. When *R*_0_ > 1, the epidemic will increase, and when *R*_0_ < 1, the epidemic will decrease.

Applications of both the theory and practice of using *R*_0_ for plant diseases, including virus diseases have been given [13,53,54,55]. As with vector-borne diseases of animals and humans, determination of *R*_0_ from theoretical models or from empirical data raises the question of how important it is to include vector-related parameters in models, or data of vector population dynamics and behaviour derived from field observations. Problems can also arise with theoretical models when host demography is included. There are no general methods for calculating *R*_0_ where there are time-dependent coefficients describing seasonality and environmental effects on host demography. However, when population demographics can be described by periodic functions the time averaged *R*_0_ may serve as a threshold for disease extinction [56]. Where host demographics are defined independently by growth functions with constant parameters, describing for example the increase and loss of susceptible hosts, then although *R*_0_ is still defined purely in terms of the epidemiological parameters, the sensitivity of the final level of infection to changes in *R*_0_ depends on the epidemiological parameter being affected by a control measure [57].

A theoretical model for the dynamics of a vector-borne plant disease led to the derivation of a basic reproduction number using the next generation matrix [58]. The value of *R*_0_ depended on acquisition and inoculation coefficients, vector mortality and birth/immigration rates, natural and disease induced plant mortality, plant recovery rate from infection, and the total number of plants in the population. The point here is that depending on the assumptions made and the parameters defined in developing the model, a different *R*_0_ may result which makes direct comparisons of the numerical values obtained problematic. Including a latent period in the model formulation adds some complexity to both the analysis and interpretation of results. In the case where new plants are introduced into the system, a proportion of these may be infected but not showing symptoms [59]. This changes the interpretation of *R*_0_. If all new plants are considered healthy, then *R*_0_ effectively sets a threshold which determines the global dynamics (if greater than 1, then an endemic equilibrium for disease persistence exists). However, if a proportion of plants are latently infected then it can be shown that a stable endemic equilibrium for healthy, latently infected and infected plants also exists.

Finally, most derivations of *R*_0_ for plant virus diseases have ignored not only the vector, but also the virus load within the plant. By making the transmission rates a function of virus titre within individual plants an expression was obtained which included within-plant multiplication in the formulation of *R*_0_. The derived expression proved valuable in determining the outcome of within-plant competition between virus strains where co-infection occurs [60], a topic discussed in more detail later. For the interested reader, there are many papers dealing with the basic reproduction number both in theory and in practice, including how it relates to the evolutionary implications [61,62,63], and to vector-borne diseases more generally where many studies continue to be published [64,65].

### 3.3. Spatial Aspects of Epidemics

An element that is receiving increasing attention is spatial aspects of plant virus epidemics, whether in terms of disease spread, spatial structure such as aggregation of host plants or disease, and the contribution of vector population dynamics, life history and behaviour to the spatial patterns of disease observed, e.g., the relative importance of seed-borne and vector transmission. Modern computational and modelling techniques make the inclusion of an explicit spatial dimension more feasible, although more demanding in terms of field observations. Three species of thrips and the incidence of tomato chlorotic spot virus infected plants were monitored regularly over three years in commercial tomato fields in south Florida [66]. The distribution patterns of both thrips and infected plants were mostly regular or aggregated with higher levels at the edge of fields which increased with time. Importantly, the study identified optimal sample sizes for a range of precision levels that could be used in disease management programs.

Having an explicit spatial basis for disease and vector surveys leads naturally to the generation of maps which illustrate the spatiotemporal progress of disease. Such maps were generated in a study of grapevine red blotch virus disease and the vector *Spissistilus festinus* at vineyards in California and New York [67]. Although there was evidence for some local spread in California, this was not observed in New York where the vector was absent. The annual rate of increase was unrelated to the estimated initial disease incidence, reinforcing the view that planting material was the initial source of virus inoculum. The preferred legume hosts of the vector tested negative for the virus indicating there was no role of these cover crops in the spread.

The spatial-temporal spread of wheat streak mosaic virus in winter wheat around a central mite-infested source of infested volunteer wheat was monitored over three successive cropping seasons [68]. Spatial gradients were determined by aerial remote sensing, ground measurements and a geostatistical technique was used to characterise the spatial pattern of disease that developed from the central source. The mapping (Figure 2 in the publication) showed that disease spread extended in all directions but with a bias related to the direction with the highest wind speeds, related possibly to wind movement of mites (not directly monitored). The study gives some information on the likely impact of volunteer wheat in fallow fields on subsequent winter wheat plantings.

Surveys are often made for the prevalence of specific virus diseases in a crop population. From an epidemiological perspective the key aspects are to place the survey design and implementation within a spatial and temporal frame. This was done for *African cassava mosaic virus* disease in Uganda during the period when there was major expansion both in the diversity of the virus species and strains, and its spread and damage [69]. Two north-south transects were surveyed for both disease incidence and whitefly vectors to investigate the dynamic nature of the disease front, from high incidence in the north to low incidence in the south. The proportion of infected plants inoculated by the whitefly vector was found to be directly related to the use of infected cuttings, stressing the need for phytosanitation by restricting the movement of planting material from affected areas and deployment of resistant varieties close to the epidemic front.

Spatial aggregation in landscape characteristics can influence epidemic dynamics but has rarely been considered in mathematical models [70]. A spatiotemporal model was developed to examine the effect of landscape patchiness on epidemic development and economic output with respect to plum pox virus disease. Management strategies were identified that performed better than current French recommendations. Although strategies were developed for each level of landscape aggregation, a strategy was also identified that was efficient at all levels and because of the simplicity of application could be deployed over a larger scale.

Spatial structure in plant populations can also affect host community dynamics through competition and other seasonal effects as well as vector movement and virus transmission. Using barley and cereal yellow dwarf viruses as an example, the effects of host community structure (perennial and annual grass species) on vector movement and disease dynamics were modelled [71]. A key finding was that connectivity of the patch structure modelled plays a major role in determining the rate of establishment of non-native species, largely through effects on BYDV infection dynamics.

Although mathematical models have been proposed to describe the temporal dynamics of plant virus diseases by means of linked systems of ordinary differential equations, the spatial dimension has mostly been ignored. The spatiotemporal aspects of disease caused by begomoviruses have been modelled [72] within and between geographical locations represented as the nodes in a connected graph. The intention was to better understand the global expansion of begomovirus disease and virus adaptability and diversity in relation to agricultural and other human-mediated practices. The modelling results pointed to the development of more diverse and less-intensive cropping patterns in time and space as the best way of avoiding the damaging effects of begomovirus disease epidemics. However, the study was limited by the lack of experimental or other data, and by its general nature in that begomoviruses affect such a wide range of crops and cropping systems.

**Figure 3:**
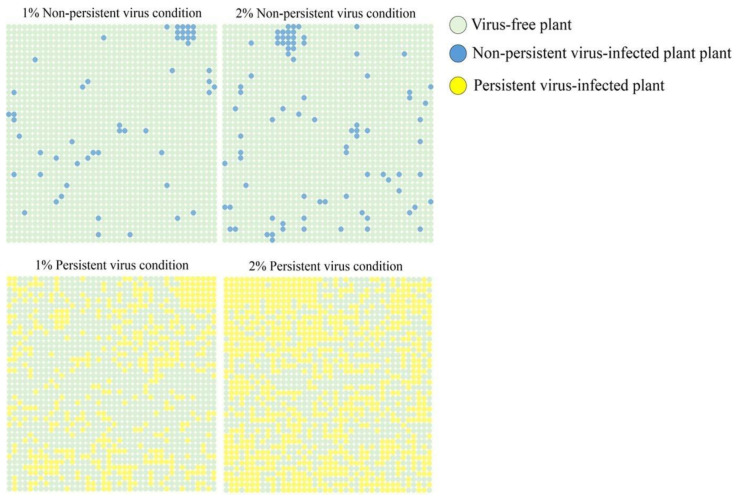
Map of simulation results for non-persistent and persistent viruses, showing the higher number of infected plants and a greater level of aggregation for the persistent virus Kho et al. 2020 [73], (graphical abstract). The 1% and 2% levels refer to the initial proportions of infected plants.

A spatially explicit individual-based model was developed to simulate the spread of persistent and nonpersistent viruses, using parameter values appropriate for potato leafroll virus and potato virus Y respectively, with *Myzus persicae* as the vector [73]. Results of simulations showed that vector numbers (both viruliferous and non-viruliferous) were marginally (x 1.3) for the persistent virus. This result was interpreted as a greater fecundity of the aphid on potato leafroll virus-infected plants. The number of infected plants was more than seven times higher for the persistent virus, interpreted as the greater opportunities for multiple inoculations by the vector, despite the period of probing and feeding on an infected plant. The spatial patterns of diseased plants that resulted at the end of the simulations showed a greater aggregation for the persistent viruses (Figure 3), interpreted as arising from the dispersal and longer retention period of the virus leading to larger patches of infection. As the authors note, this distinction between the two transmission types is discrepant with some field studies, largely because the simulation model they use is concerned with a single vector species and local spread, whereas multiple vectors with alate forms which can be colonising or non-colonising may be involved in field spread.

Where virus disease has caused the widespread destruction, deterioration or abandonment of plantings, whole areas may be cleared to enable the planting of healthy, possibly more resistant material. Such a situation occurred in Ghana with cacao, where because of the widespread damage caused by cocoa swollen shoot disease, new block plantings of more resistant cacao genotypes were made. Virus spread within the crop can take place by radial movement from the periphery of the block plantings adjacent to previously infected, possibly relic, populations of cacao; or by wind-blown ‘jump spread’ into the interior by the relatively immobile mealybug vector. Given these two scenarios of spread, the effectiveness of imposing a cordon-sanitaire around the plantings was modelled using a spatiotemporal model [8,74]. Although estimates of the frequency of jump spread are rare, and mostly obtained from mealybug trapping over open water, it seems to have less impact on the rate of re-introduction of the virus than radial spread from the perimeter of the planting. A cordon-sanitaire markedly delays the rate of re-introduction, but eventual control would depend on the level and durability of resistance and whether rogueing of diseased plants was practiced, elements which were not modelled.

### 3.4. Environmental Drivers

**Figure 4:**
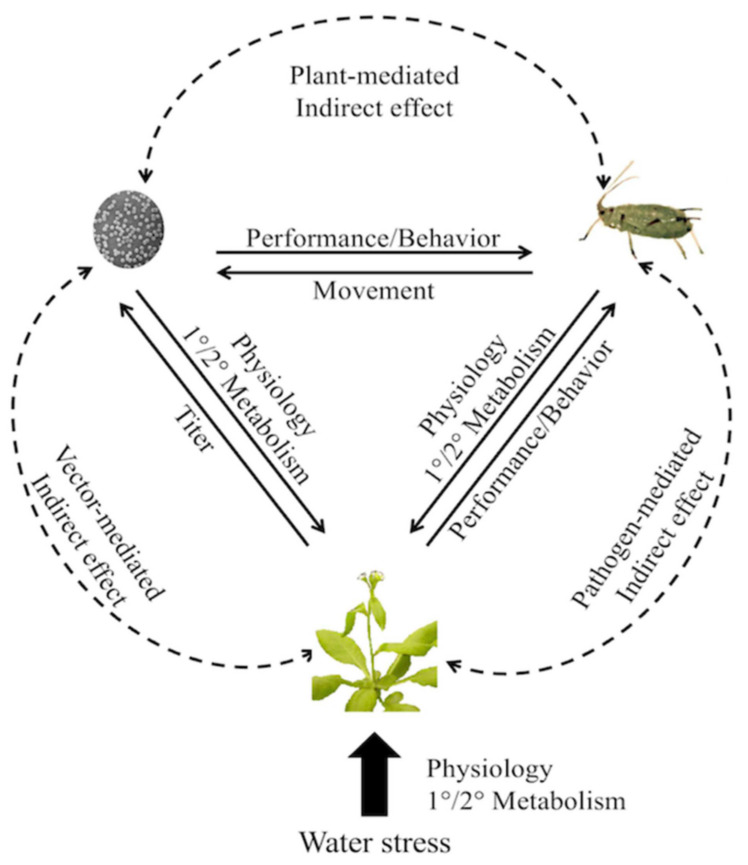
Direct and indirect effects arising from interactions between vectors and viruses on drought stressed plants, with directionality shown by the arrows, Szczepaniek & Finke, 2019 [77], (Figure 1). Both the vector and the virus induce a direct physiological response from the plant. The plant imposes direct effects on virus titre and on vector performance and behaviour.

Many reviews have dealt with the effects of climate change on plant pests and pathogens, particularly in relation to global warming, but to some extent plant viruses have been poorly represented [75]. For example, an overview [76] of 75 review papers on this topic published during the period 1988–July 2019, reveals that only six were concerned explicitly with plant viruses, although arthropod vectors were included and could be added to this number. Plants are subject to biotic stress arising from drought, exacerbated by the global increase in drought events arising from climate change. The whole plant response to drought inevitably leads to physiological changes that affect the response to biotic stresses such as those posed by pathogens. In the case of vector-borne diseases, there is then a tripartite relationship that needs to be considered if the impact of climate change is to be fully appreciated [77] as shown in Figure 4. In this review, many examples of host plant–virus–vector associations affected by drought are presented, including viruses transmitted by aphids, whiteflies and thrips.

The impact of abiotic stresses on plant virus transmission and virus spread was recently reviewed [78]. Elevated temperature, CO_2_ concentration, drought and flooding may be expected to have effects on host responses and emphasises the need to consider both biotic and abiotic constraints, and their interaction on plant virus epidemiology. As well as having effects on virus transmission, environmental factors can also affect tripartite and by implication tritrophic interactions [5]. Environmental effects can influence the severity of virus infection. With infection of wheat by barley yellow dwarf virus, it was found that the timing of water stress was important in affecting plant performance [79]. When virus infection preceded periods of water stress, plant performance was not reduced, and infected plants recovered sooner than non-infected plants. However, vector preference for feeding on infected plants lead to greater herbivory, although the effect on fecundity was more pronounced with low rather than ample water supply. A recent study suggested that drought can lead to a transition from parasitism to mutualism [80].

A further abiotic stress, which by comparison with water or temperature stresses has been little studied is that imposed by light intensity and quality. This aspect was studied in a recent study which looked at the effect of light intensity on seed transmission of viruses [81]. The hypothesis tested was that light conditions which favour within-plant multiplication also favour seed transmission, using *Arabidopsis thaliana* challenged with either turnip mosaic virus (TuMV) or CMV. The hypothesis was supported with TuMV; but with CMV, higher light intensity reduced CMV multiplication and had no effect on seed transmission.

A key issue in determining the effects of the environment, is noting the difference between weather (often local and immediate) and climate (regional and seasonal) and how the associated meteorological variables can be used in forecasting and prediction of plant virus epidemics. Weather, or current meteorological conditions, can be used as part of a decision support system to help identify risk factors that can inform disease management decisions. One of the most developed for plant virus diseases is Peanut Rx, in which an assessment of relative risk of tomato spotted wilt disease is made available to peanut growers. The principal vector of *Tomato spotted wilt virus* (TSWV) in the south-eastern USA is *Frankliniella fusca*. Recently, the influence of meteorological factors on thrips flight and dispersal was incorporated as an add-on to Peanut Rx [82]. When heat sum accumulation and a precipitation index were incorporated into Peanut Rx, 79% of high-risk instances were predicted rather than 56% without meteorological data. Although the false-positive rates were high in both cases, this was considered reasonable given the inbuilt bias of the system in relation to uncertainty in the risk assessment. Weather variables were also used as predictors for both cotton seedling susceptibility to thrips infestation and thrips generation times [83]. Combining these two aspects gave a model for seedling damage that was further developed as software for a prediction tool. This approach would be entirely appropriate for thrips transmitted viruses of other crops.

Inadequate nutrition can also lead to abiotic stress. Although accounting for nutrient supply is an essential feature of crop modelling, particularly for nitrogen and phosphorus, there have been few accounts in relation to modelling plant virus disease dynamics. A plant-growth model based on physiological processes was combined with a pest population-dynamic model and applied to the green peach aphid system in terms of direct herbivory effects but also indirectly for virus transmission [84]. The aim was to distinguish between the plant vigour hypothesis, in which the aphid population would increase most rapidly on vigorously growing plants, where nutrition and water are not limiting, compared with the plant stress hypothesis, in which aphid populations would increase more rapidly on stressed plants, where resources for plant defence are depleted but nutritional quality may be enhanced. It was found that there was no simple support for either hypothesis as the outcome depended on the timing and levels of fertilisation and irrigation. Although virus transmission was not modelled explicitly there are clearly parallels with how aphid behaviour and performance could lead to different epidemiological outcomes.

Two models differing in the way nutrient supply affects disease dynamics were developed and tested against data on virus accumulation of cereal yellow dwarf virus and number of infected phloem cells from stems of *Avena sativa* [85]. Uniquely for models of plant virus dynamics, a basic reproduction number was derived depending in part on nutrient-mediated virus production parameters.

### 3.5. Production Systems and Cycles

Many important food crops are propagated vegetatively, e.g., sweet potato from vines and cassava from stem cuttings. In these production cycles, the planting material is often taken from farmers’ fields or through informal exchange with other farmers. In such systems, viruses can multiply and intensify leading to progressive degeneration in the varieties grown. However, a phenomenon known as reversion has been reported in which plants propagated from previously virus infected plants are free from virus [86], although this can depend on variety and whether the plants were infected with single or multiple viruses. The reversion phenomenon can in principle be used in breeding programmes and as a component of disease management through varietal deployment.

Farmers’ fields are generally part of a wider environment which includes wild plants such as agricultural weeds within the crop or natural flora in field margins, abandoned fields or in land at urban margins. Many studies have recognised the interface between crops and the wider environment as important in the interaction between plants, vectors and viruses, often influencing virus transmission and disease spread at a local level. A fewer number of studies have attempted to extend the analysis globally. An example is given for the Solanaceae family, which has species in all the settings above, is present globally, and is affected by a wide range of viruses [87]. The authors concluded that new disease management practices and diagnostic methods would be needed to cope with the global changes affecting the agricultural–environmental interface, by targeting the entire *Solanaceae* community.

The use of insect screening to prevent the entry of virus vectors is practiced in plant nurseries and in field cultivation for some horticultural crops. Experiments were designed to reduce the entry of *Bemisia tabaci* into tomato crops by insect screens, with or without insecticide-treated strips [88]. A mathematical model was developed using symptom data of tomato yellow leaf curl disease and potato yellow mosaic disease. Parameter estimates using the model fit indicated that screening reduced vector immigration by about 12%, but also increased slightly the retention within screened plots, even with mortality caused by the insecticide strips. Without insecticide strips, there was a large increase in vector retention within the screened plots leading to a greater disease incidence than in control non-screened plots.

In annual production cycles, the time gap between harvest and planting the succeeding crop may be short, with obvious implications for the carry-over of virus and vectors. A model for this scenario looked at repeating production cycles [89]. Parameters of the model were considered appropriate for a whitefly-transmitted carlavirus of soybean, although the model was quite generic. Analysis of the model revealed a threshold vector population size that determines whether, or not, the disease goes to extinction over successive production cycles. The emphasis is placed on the final size of the epidemic in relation to the final size at the end of the previous production cycle, rather than on conditions at the start of an epidemic. In principle, this threshold result could be used to evaluate the extent to which vector control measures could be used in disease management without the need to eradicate the vector.

### 3.6. Phytosanitation and Rogueing

Rogueing of diseased plants has long been practiced as a means of disease control, sometimes with the aim of eradication but more frequently either to contain an outbreak, or to maintain disease levels below economically damaging levels. Rogueing of diseased plants is often practised in combination with their replacement by healthy stock, especially for perennial tree fruit crops. An early model for plant virus disease dynamics was developed primarily to determine the effectiveness of rogueing of diseased with their replacement by healthy plants [90] in order to maintain a constant population size. The basic reproduction number was determined and included terms for continuous rogueing and replanting. From these expressions, the likelihood of rogueing and replanting being effective for four virus diseases, citrus tristeza, banana bunchy top, cocoa swollen shoot and plum pox was evaluated using parameter values estimated from literature. The main conclusions were that: (a) at low contact (transmission) rates, rogueing when plants are infectious can be effective in eradicating the disease (over a period of time); (b) at high contact rates, rogueing latently-infected as well as infectious plants would be needed; (c) at high replanting rates, the disease is more difficult to eradicate, leading to a trade-off between rogueing and replanting to achieve optimal control.

This model was further developed with a more realistic representation of rogueing and replanting as periodic pulses rather than continuously [91]. Although an explicit solution could not be found for *R*_0_, its value could be calculated numerically from parameter values. Some important findings were found: (a), when the infection rate is high it may be impossible to eradicate disease by rogueing only infectious plants and so identifying latently infected plants would be key; (b), increasing replanting rates is counterproductive for disease control; and (c), the model of Chan & Jeger [90] with continuous rogueing may overestimate the infection risk (*R*_0_ > 1) compared with pulse rogueing (*R*_0_ < 1).

The effectiveness of rogueing was modelled for banana bunchy top virus and predicted to be achievable [90]. Two key features of a banana bunchy top epidemic were subsequently modelled: temporal increase over a 10-year period and the gradient in disease from the edge of a plantation [92]. Different strategies of disease control were then explored to determine the most effective methods of rogueing but also the risks associated with these methods.

Cassava is a crop in which both rogueing of plants infected with cassava mosaic disease and subsequently replanting with new stem cuttings taken at harvest are practiced. In some circumstances, farmers are faced with the choice of stem cuttings taken from healthy or infected plants, based on the availability of planting material and the phenomenon known as reversion in which some cuttings taken from infected plants can lead to healthy plants. Such a choice may depend on farmer preferences, and the consequences of such preferences have been modelled [13,93]. In this model, choice was based on the frequency of infected and healthy plants, according to a selection coefficient (reflecting farmer preference). More recently, a model for replanting was proposed in which choice was based not on relative frequency but on the population abundance of infected cuttings, with a weighted coefficient against their use [94]. Optimal control strategies were determined for both models. It was found that a greater control effort (using rogueing and vector control) was required to eradicate the disease at low levels of infection with the abundance model than with the frequency-dependent model. However, with a high frequency of use of infected cuttings, unanticipated outcomes may result when controls are applied, making the choice of replanting strategy (even if made unconsciously by farmers) an important issue.

There are examples of other vegetatively-propagated crops where there is a lack of separation between the plantation crop and the production of planting material, especially in less developed informal systems of production. Material may be recycled from the plantation or mother trees to produce the next generation of planting material, thus perpetuating the presence of viruses even where there is a level of reversion to the healthy state. The outcomes in a model describing rogueing and replanting in such a combined system [95] could be: (a) 100% disease saturation; (b) a situation in which both diseased and healthy plants persist; and, importantly, (c) a criteria which determines whether healthy plants can ‘re-invade’ a completely diseased plantation.

Rogueing with annual food crops gives less promising results. Data from 2-years of field trials at the International Rice Research Institute in the Philippines were used as a comparison with simulated results from a model for rice tungro disease (RTD) [96]. The model was based on published information on rates of virus transmission, vector population dynamics, and vector dispersal. The model was then used to evaluate the effectiveness of rogueing diseased plants and replacing them with healthy plants in preventing further disease spread. It was found that when disease levels were relatively high, simulated rogueing was ineffective even when carried out efficiently. When disease incidence was low, rogueing although effective was of little consequence. However, there were no rogueing interventions made in the field trials, although other unpublished observations in the region suggested that disease incidence could be reduced, but not significantly where there is a low prevalence.

### 3.7. Host Resistance Deployment

Breeding for disease resistance plays a major role in developing strategies for disease management. This is especially the case for plant virus disease, but as with other pathogen groups less attention has been given to how host resistance should be used in the field. A large body of work has been directed at the development of plants with resistance to plant viruses, combining the screening of molecular markers for genotype selection through to phenotype selection in field trials. However, by comparison little has been done on the transmission and spread of plant viruses in resistant varieties under field conditions. From an epidemiological perspective, this is a major gap that is holding back progress in disease management and will be emphasised in this section.

Potato virus Y (PVY), although long-known as a major potato pathogen, has emerged recently as a range of strains that challenge potato production globally [97]. Taken together, properties of the virus, its response to deployment of resistance genes, and vector relationships, and how these come together in an epidemiological framework, offer the best clues to disease management but it remains unclear how widely these can be applied across different geographical locations, potato cultivars, and strains of the virus. Epidemics of sweet potato virus disease (SPVD, caused by sweet potato feathery mottle virus and sweet potato chlorotic stunt virus) were monitored over time and with leaf profile for 10 sweet potato varieties covering a range of resistance characteristics [98]. The data provided detailed information on the development of SPVD and the molecular responses that were occurring in the field based on transcriptomics. It was found that resistance was characterised both by disease incidence on a per plant basis, but also by the number of symptomatic leaves per infected plant. Such an approach can provided invaluable information to guide molecular breeding approaches, and further how the developed varieties can best be deployed in the field.

Vector feeding resistance might be expected to reduce incidence and the spatial distribution of virus disease in crops. The spread of bean pod mottle virus (BPMV) was monitored in resistant compared with susceptible soybean [99]. Although a spatial aggregation of disease was found, this did not depend on host genotype and resistance per se was insufficient to reduce disease incidence and spread. In another study, resistant and susceptible genotypes of groundnut were evaluated in thrips choice and no-choice feeding tests [100]. Fewer adults and larvae and less feeding damage were found on the TSWV-resistant variety Tifguard than on the susceptible genotype Georgia Green, but not necessarily on other resistant genotypes, indicating that observed field resistance in this genotype may result from the interaction between the thrips vector and groundnut genotype.

Tolerance as a disease management strategy has been claimed to be as widespread as host resistance although problems remain in the strict definition of tolerance and how it can be assessed. For some workers, especially those concerned with crops, it refers to limited symptom development or reduction in plant vigour or yield in a cultivar despite a normal virus accumulation that would be expected in a susceptible cultivar. For other workers, more concerned with the ecological and evolutionary aspects of plant-virus interactions, tolerance would be measured as the limited reduction in plant fitness (fecundity, reproduction period). In a resistant (not immune) variety, there would be limited virus accumulation and symptom development, although there may be a penalty in terms of reduced vigour and yield in the absence of disease compared with a susceptible variety. Whether there are trade-offs in tolerances to different viruses was studied recently in *Arabidopsis thaliana* genotypes challenged with either CMV or TuMV [81]. It was found that tolerance to CMV was associated with resource allocation from growth to reproduction, whereas for TuMV it was associated with the time to and length of the reproductive period. There was a genotype-dependent trade-off in tolerance between the two viruses. This finding carries implications for disease management based on tolerance when more than one virus is present in a crop.

Novel ways of improving the resistance characteristics of crops by exploiting microbial interactions can also assist in disease management, but these have not received the same attention or funding given to plant breeding. Pre-treatment of grain with the plant growth promoting fungi *Penicillium simplicissimum*, *Fusarium equiseti*, and *Penicillium asperellum* induced resistance in faba bean mechanically inoculated with bean yellow mosaic virus [101]. Disease severity and virus titre were significantly reduced by singly applied treatments with a significant increase in expression of pathogenesis related genes compared with non-treated plants. There was a strong improvement in faba bean growth characters. No indication was given of how induced resistance techniques could be developed and applied in field settings. Arbuscular mycorrhizal fungi form mutualistic associations with most terrestrial plants, improving plant performance, water and mineral uptake, and providing a level of protection against abiotic and biotic stress, including against plant pathogens. There have been fewer reports of beneficial effects against plant viruses, compared with fungi, bacteria and nematodes. In glasshouse experiments, a significant reduction in disease severity and accumulation of tomato mosaic virus was found in mycorrhizal tomato plants as well as the enhancement of quantitative and qualitative plant growth characters [102].

### 3.8. Crop Heterogeneity

The impact of biodiversity on plant pests and diseases continues to evoke considerable interest both from its intrinsic value and from the contribution it can make to pest and disease control. The mechanisms of such control have been much researched with a consensus reached that biodiversity among potential plant hosts, within or between species, provides a dilution effect that limits the impact of pests and pathogens. A major meta-analysis has estimated the size of the dilution effect, as represented by species richness metrics, for both fungal and viral pathogens [103]. They found that a strong dilution effect was found for obligate biotrophs (by definition, this holds for viruses), but not for necrotrophs. A further argument for the deployment of crop diversity is that it slows down or restricts the evolution of pathogen populations which have matching pathogenicity to the crop genotypes being deployed. The evidence of this for plant viruses is unclear. It has been reported that in experimental populations of *Arabidopsis thaliana* with serial passaging of TuMV, the evolving isolates were more pathogenic in heterogeneous populations than in a metapopulation composed of distinct subpopulations [104].

The use of varietal mixtures where the individual varieties differ in their resistances to pathogens has long been studied as a strategy to exploit crop heterogeneity. Such an approach has also been proposed for insect pests, such as aphids, where genetic isolation and adaptation during the parthenogenetic stage are well known. A modelling approach [105] was taken in which the effect of including aphid-susceptible varieties would slow down the development of resistance-adapted aphid genotypes. Based on deterministic modelling results, a threshold was determined for the initial frequency of resistance-adapted genotypes below which such genotypes would be eliminated even if the non-adapted genotypes had no selective advantage in fitness. The most important parameters in the threshold, confirmed in a stochastic version of the deterministic model, were the frequency of susceptible varieties in the mixture, the level of aphid resistance in the resistant varieties, and the aphid growth rate. These results need careful consideration in modelling how variety mixtures, or other forms of crop diversification, would affect virus diseases vectored by aphids.

Much of the work on varietal mixtures has been done within a cropping season. The longer-term dynamics of disease in a dynamic cropping system, with deployment of virus-resistant and susceptible varieties and other transitory crops has not been looked at in the same detail. In rice cropping systems, there can be a range of crops of different growth stages grown in the same locality. RTD can persist in a locality with the movement of virus from crops. Using a mathematical model of RTD, the effect of planting resistant varieties as a component of the cropping system over time was investigated [106]. Provided that the deployment of resistant varieties was spatially random, then a logarithmic relationship was found between the proportion of susceptible crops and the spatiotemporal spread of disease. However, a large proportion of fields needed to be planted with resistant varieties in order to have area-wide impact and reduce virus disease incidence in fields of susceptible varieties. In the case of two rice crop seasons per year, the recommendation was to grow the resistant varieties in the season of highest risk of spread; this was a better option as growing them in the season of lowest risk to prevent carry over had little effect on subsequent spread.

Intercropping with a range of geometric arrangements of unrelated host plants is a more general form of crop biodiversity than the use of intraspecific varietal mixtures. There have been many studies on intercropping and insect pests but fewer on virus vectors and virus disease. A model was developed that monitored aphid and virus spread in an intercropped field, the spatial arrangements of intercropped plants, and the use of trap plants within the intercrops [107]. Contrary to current practice it was found that a ‘chessboard’ arrangement of plants was a better option than row or strip intercropping in terms of reducing the number of infected plants, the rate of increase in disease across the field, and the derived basic reproduction number. This result was obtained with or without the use of a trap plant within the intercrop. Intercropping could also lead to evolutionary change in a vector population, as shown in a model developed for *B. tabaci* [108], although the attractiveness of trap plants was more critical than intercropping per se, and the time horizon for change was not specified.

### 3.9. Combinations of Disease Control Measures

Integrated pest management has become the gold standard in control for many insect pests but has not always been accepted by plant pathologists [109]. However, for those pests which are virus vectors, IPM programmes have had greater acceptance, such as with Western Flower Thrips, *F. occidentalis* [110], As such, plant virus control of tospoviruses falls within the IPM paradigm [111]. Single disease control measures for plant diseases are seldom sufficiently effective when practised alone, especially for plant virus diseases. Often host plant resistance is claimed as the main long-term solution, but deployment of such resistance must fit in with other crop protection and cropping system practices. Combining host resistance to *African cassava mosaic virus* with natural escape mechanisms, “reversion” and cutting selection was modelled to determine the effects on disease incidence and yield losses [112]. The results strongly supported the use and integration of host resistance and phytosanitation measures, even with high whitefly vector populations. The need for integrated disease control measures for begomoviruses, based on cropping system, host resistance and phytosanitation was stressed [113], particularly concerning the cassava mosaic disease epidemic in East Africa.

Degeneration of vegetative planting material can occur rapidly in circumstances where virus-free planting material is unavailable or too expensive in resource poor counties with limited farmer support and logistical services. A risk assessment framework was developed to model how best to develop an integrated strategy involving on-farm selection, assessing the extent of external sources of virus and the risks posed, reducing the rate of within-field transmission, and combining different control options by making use of host resistance to reduce the need for vector control [114]. Such integrated steps keep degeneration below a defined threshold and extends the time before renewal with certified planting material becomes necessary to maintain yields.

## 4. Transmission

In the main plant viruses are transmitted horizontally by arthropod vectors, or vertically through true seed and vegetatively-propagated planting material. These cases and their interaction are the main emphasis in this review, but it should also be recognised that nematodes and fungi also transmit viruses. There are also relatively unexplored pathways of plant virus transmission in relation to disease epidemiology, including direct contact, root grafting, parasitic plants, and contamination of soil and water [115], but these are not considered here.

Transmission will be reviewed in four main areas in this section:

(i) horizontal transmission by arthropods;
(ii) vertical transmission;
(iii) interactions between horizontal and vertical transmission;
(iv) transmissibility, virus accumulation and virulence

In subsequent sections, three key areas relating to vector transmission will be discussed:

(v) the effects of vector population dynamics, behaviour and feeding, and how these are affected by natural enemies;
(vi) the conditional vector preferences for infected or healthy plants;
(vii) the common occurrence of coinfection of plants by multiple virus species, strains or genomic segments.

### 4.1. Horizontal Transmission by Arthropod Vectors

**Figure 5:**
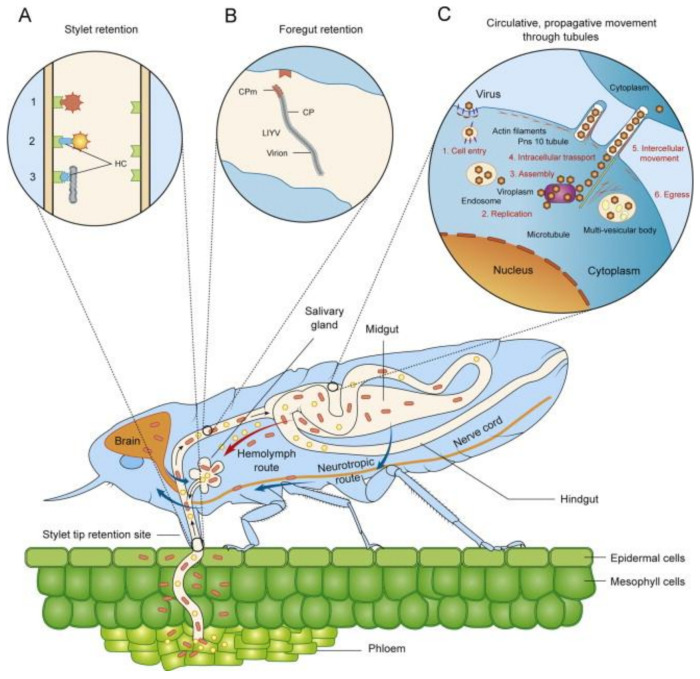
Schematic showing how the retention and movement of plant viruses leads to classification of transmission mode, Whitfield et al. 2015 [120], (Figure 1). In this representation, the classification is made in terms of: A stylet retention (elsewhere described as non-persistent), B foregut retention (semi-persistent), and C circulative movement (including both persistent-circulative and persistent-propagative.

Transmission is a continuum of processes starting with acquisition of virions when a vector probes/feeds on an infected plant, passaging and retention of the virions at specific sites in the vector, and subsequent inoculation of a recipient plant [116]. Each of these processes is probabilistic, dependent on virion survival, and vector life cycle and behaviour [117,118]. There have been many overviews of virus transmission of by arthropod vectors, each with different emphases: comparisons between plant and animal viruses [119], comparison across transmission types [120] (Figure 5), specific transmission types [116,121,122,123], vector taxonomic groups [124], and virus taxonomic groups [125,126].

An understanding of transmission has claimed as necessary for determining disease control strategies [120,127]. A comprehensive review of plant virus transmission by all classes of known vectors has been made with an emphasis on how innovative control measures can be developed [127]. An equally innovative scheme was proposed for classifying vectors (but not including hoppers or mealybugs) in terms of mode of transmission, the relative timeframes for acquisition, retention and inoculation, and the structural elements and mechanisms involved, and how this classification relates closely to different virus groups. A relatively unexplored pathway of transmission is sexual transmission through vector. Tenuiviruses can be transmitted transovarially by viruliferous female planthoppers to their offspring, and through sperm from viruliferous males [128]. Experimental [122,129] and modelling studies [130] have explored the possibility of sexual transmission and its potential significance in disease epidemiology.

Plant viruses in the main are acquired by vectors feeding on the phloem, although acquisition of nonpersistent viruses occurs during probing without feeding. Some insects, including cicadellid leafhoppers also feed from the xylem. The glassy-winged sharpshooter *Homalodisca vitripennis* is a vector of the xylem-limited bacterium *X. fastidiosa*, but virus sequences have been found in this species that are closely related to the plant reovirus rice dwarf virus, a virus that is not limited to the phloem [131]. Although possibly exceptional, this example makes the point that acquisition may occur in ways other than those seen as the norm. It is also known that TuMV moves systemically to vascular tissues through the phloem; and, also, via the xylem, although the evidence for de novo RNA synthesis is indirect [132]. Insects that are predominantly phloem-sap feeders also consume xylem sap for reasons of restoring water balance following dehydration or other osmotic stresses [133]. Finally, on this topic, it seems that the feeding behaviour of some cicadellids such as the beet leafhopper *Circulifer tenellus*, although a phloem feeder, does not achieve the high rate of ingestion typical of other phloem feeders [134], although the implications for transmission of beet curly top virus are not drawn out.

The persistent transmission of viruses and the implications for insect-virus interactions has been reviewed comprehensively [121] and specifically for virus groups within this class [125]. One possibility is that these plant viruses may have originated as insect viruses, especially those known to propagate, move transtadially and transovarially. In that sense the plant may be considered as the vector allowing the virus to be transmitted between insects [130,135]. Examples of this may be seen for tospoviruses, belonging to the virus order Bunyaviridales, which mostly includes animal viruses, and plant infecting members of the Reoviridae. What needs further investigation from an epidemiological perspective is the effect of the virus on the insect vector and whether there is a trade-off between persistent transmission and any fitness cost to the vector [136].

The form of transmission by vectors is arguably the key epidemiological characteristic of plant virus epidemics, as it defines the temporal scales of acquisition, retention, inoculation, and vector life history and behaviour that determine the rate of disease increase. A theoretical SEIR model was developed which described epidemic processes for the main classes of transmission: nonpersistent, semipersistent, persistent-circulative, and persistent-propagative [137]. The basic reproduction number and the final epidemic size were derived in terms of parameters appropriate for each transmission class. A more comprehensive analysis of the effects of vector mobility migration was made using numerical methods [138]. Compared with the non-persistent class, at low insect population densities and in the absence of net immigration, there was a greater disease development for the semipersistent and persistent-circulative classes using representative parameter values. Changes in vector longevity affect most the persistent-circulative and persistent-propagative classes, whereas the latter class was least affected by vector mobility within crops. When vector migration was explicitly considered, the outcome depended on the proportion of infectives in the immigrant population, and the proportion of emigrants (or those dying) replaced by immigrants. The persistent-propagative class was highly sensitive to changes in the balance between these two factors. These results demonstrate that vector related factors, other than acquisition and inoculation rates, affected the transmission classes in distinctive ways and have a major impact on disease dynamics.

Transmission of plant viruses can affect plant virus epidemiology in many ways other than disease dynamics. Within-host and between-host population bottlenecks in transmission have been documented but estimates of their size are rare [139]. The emergence and extinction of plant viruses depends on the bottlenecks associated with transmission events [140]. In an experimental study with aphid-transmissible strains of PVY, and using stochastic estimation methods, it was found that the vector *M. persicae* transmitted many fewer virus particles (<5) per inoculation (following acquisition) than the census virus population would suggest. These results indicated that genetic drift may have a major effect on plant virus populations during vector transmission.

Many models of plant virus epidemics assume that vector transmission takes place without regard to spatial heterogeneity of vectors within crops or on plants. A model of the effect of spatial aggregation of vectors on disease dynamics [141] was developed due to the lack of fit obtained with a conventional bilinear representation of transmission (contact between healthy plants and infective vectors during inoculation and between non-viruliferous vectors and infected plants during acquisition) with regard to field data on whitefly transmitted African cassava mosaic disease. Incorporation of an aggregation term in the model allowed a much-improved fit and suggested that such aggregation could reduce the infection rate in some circumstances. It should be noted however that the model did not consider vector preferences as discussed in later sections. Many studies have shown that wild plants and crop weeds can serve as alternative reservoir hosts for viruses infecting crop plants [142]. It is important for epidemiological reasons that experiments designed to show this should be done with the natural vectors of the virus. The mite vector of citrus leprosis citrus C was raised on fruits of infected sweet orange and then transferred to plants of four hedgerow plant species commonly present around citrus groves [143]. Descendent mites were then transferred back to healthy sweet orange after some three months to determine whether the virus was transmitted. Typical symptoms of citrus leprosis virus were subsequently observed in most of the citrus plants tested. The implications of this study are that the presence of such plants in the vicinity of a citrus grove may serve as hosts for the mite and for the virus, and hence a reservoir for virus spread into citrus, although as noted by the authors such an outcome needs testing under natural conditions.

### 4.2. Vertical Transmission

Vertical transmission of plant viruses through seed has been less studied than horizontal transmission through vectors. As well as the better-known transmission through seed or pollen, another form of vertical transmission can occur by integration of viral genomes into the host genome [144]. The epidemiological implication of this form of vertical transmission have received little attention, although for banana streak virus the interaction with the mealybug vector has been documented [145]. Seed transmission, either through infection of the seed coat or of the embryo is well documented often using long-established laboratory seed testing methods; sometimes using mechanical transmission methods [146,147], sometimes through field samples. Similarly, methods for growing-out plants from infected seed have long been practised [146,147] to assess the transmission from seed to the developing seedling.

The various stages of seed transmission that are relevant epidemiologically are the movement of virus from mother plant tissues to seed, from the embryo to the progeny seedling, and the contribution of seed transmission to local and long-range dispersal of viruses. More specifically [148], the efficiency of seed transmission would be determined by: (a) virus within-host multiplication and movement, (b) the ability of the virus to invade gametic tissues, (c) plant seed production upon infection, and (d) seed survival in the presence of the virus. In some instances of severe infections, seed transmission may be possible, but seed may not develop fully due to severity of the infection. These predictions were supported by estimates made in experimental work by the authors with *Arabidopsis thaliana* and TuMV and CMV.

The epidemiological significance of seed transmission is that even at low levels of seed transmission, this can be amplified by subsequent vector transmission, and hence can be responsible for the introduction of virus into new areas and trigger epidemic development [149,150]. Seed transmission is also of ecological significance in that it allows virus survival between growing seasons. Some viruses appear to be transmitted exclusively through seed or pollen, and such transmission may place limits on the host range of a virus, with separation of isolates according to host [151]. Epidemiologically, a major population bottleneck imposed on genetic diversity may occur in virus transmission from seed germination to seedling growth [152].

### 4.3. Interactions between Horizontal and Vertical Transmission

Seed transmission can also accompany transmission with vectors, including the plasmodiophorid *Polymyxa* [153] and mealybugs [154]. A similar situation can occur when pollen transmission can accompany vector transmission [155]. The related issue of virus transmission through pollen offers a mechanism for both vertical transmission to progeny, and horizontal transmission to the same cohort of plants [156]. Grow-out experiments have also shown to be necessary to estimate pollen transmission rates [157]. In some cases, it has proven difficult to disentangle pollen transmission from direct maternal transmission to seed [154].

In the context of whether transmission is horizontal or vertical, or a combination of both, the effects on pathogenicity and virulence can be compared. This was explored in a theoretical model [158] raising the question whether high levels of vertical transmission with low virulence could be observed. It has been shown using a model with two parasite strains that a vertically transmitted strain of an endophytic fungus of grass that would go extinct on its own, can co-exist with a horizontally transmitted strain [159]. It is also the case that models suggest that transmission-virulence trade-offs can lead to mutualistic rather than parasitic relationships [30]. An interesting insight that mode of transmission can lead to evolutionary bistability was developed for a fungal disease of wheat [160]. The implications are that both vertical and horizontal transmission may be stable traits, depending on the ecological circumstances. The question that then arises is whether there is evidence for a switch between vertical and horizontal transmission for plant viruses.

### 4.4. Transmissibility, Virus Accumulation and Virulence

Blanc [161] suggested that transport of viruses within plants and vector transmission between plants may be closely linked. Two relatively unexplored concepts at that time required further research: firstly, that virus accumulation and structural complexes within plant cells, independently of movement between plant cells, may facilitate virus acquisition by vectors; secondly, that a virus successfully replicating in a plant cell may initiate mechanisms that either permit or prevent the probability of multiple infections by related or unrelated viruses.

Virulence is defined as “the degree of damage caused to a host by parasite infection assumed to be negatively correlated with host fitness” whereas “pathogenicity is the qualitative capacity of a parasite to infect and cause disease on host” [162]. These definitions, although entirely consistent with definitions in evolutionary biology and ecology, could almost be switched in plant pathology with groups other than viruses, especially fungal pathogens, where virulence is a qualitative trait and pathogenicity a quantitative trait. This divergence can lead to mutual misunderstandings between plant pathologists concerned with different pathogen groups, and even among epidemiologists. According to these authors [162], selection acts on both traits and despite much theoretical analysis, experimental work has continued to diverge and the differences between viruses and fungi as plant pathogens continues to be exacerbated by the different perceptions and terminologies used by the two groups of plant pathologists.

The reduction in host fitness caused by a pathogen or parasite, has long been a concern of evolutionary biologists, especially as to whether there is a trade-off between virulence and transmissibility. For plant viruses, although most are horizontally transmitted by vectors, there are also routes for vertical transmission through seed as discussed above. Despite much theoretical modelling, there have been few experimental attempts to disentangle the relative effects of horizontal and vertical transmission on the evolution of virulence. The evolution of CMV on *Arabidopsis thaliana* was studied by serial passaging of different CMV strains under either horizontal or vertical transmission and then alternating the mode of transmission [163]. Passaging through solely vertical transmission led to higher rates with time but also lower virus accumulation and virulence. There was also host adaption to vertical transmission that amplified these effects. Solely horizontal transmission had no significant effects on either of these traits.

The relationship between virulence and transmissibility remains a topic of much interest. From a theoretical perspective, increased virus transmission should be correlated with increased pathogen-induced mortality (and reduced period of being infected), because both variables are functions of within-plant virus accumulation. However, it has been pointed out [164] that there are few experimental studies that support a positive correlation between plant virus accumulation and virulence. Therefore, although most studies confirm a positive correlation between within-plant accumulation and transmission, strategies to test for a trade-off, a level of virulence that would optimise transmission from an epidemiological perspective, have yet to be developed for plant viruses. The relationship between virus accumulation and virulence may be more complex when two competing strains are present [165].

## 5. Vector Behaviour

Information on the general biology and ecology of arthropod pests is essential for effective pest management. This applies in terms of their direct effects on crops (e.g., [110]), indirect effects on secondary pests, and the viruses and other pathogens they may vector.

### 5.1. Population Dynamics and Dispersal

The most efficient vector is not necessarily the most relevant epidemiologically. This depends on vector abundance and seasonality at times when host plants are most susceptible, as found for plum pox virus and its aphid vectors on peach [166]. Incorporation of vector population dynamic and life history parameters into models of virus disease dynamics, is essential both for understanding and prediction of epidemics, especially where the virus affects these parameters. Increasing life expectancy of the thrips *F. occidentalis* on tomato spotted wilt infected plants promotes multiplication and spread of the virus in a crop [167]. However, the level of resistance or susceptibility to TSWV in crops such as groundnut can affect thrips feeding and survival, which were reduced in some TSWV resistant genotypes, with consequent reductions in virus infection and accumulation [100]. Insect vectors often have a greater survival and reproduction on virus-infected plants. For example, infection by tomato yellow leaf curl virus promoted survival and reproduction largely through the virus C2 protein in begomoviruses lacking DNA satellites suppressing plant defences by interacting with plant ubiquitin [168].

Vector life history and behavioural parameters play a key role in virus transmission and disease spread. Although not always considered explicitly, there is increasing interest incorporating them into models of disease dynamics. In many models the assumption is made that birth and death rates are the same for viruliferous and non-viruliferous vectors. This assumption was modified by allowing the per capita death rate of inoculative vectors to be increased relative to non-viruliferous vectors, and similarly for birth rates, and comparing the outcomes to the standard model [169]. It was found that the effect of increasing the death rate of infective vectors reduced vector population density, the proportion that was infective, and disease incidence. Varying the fecundity of infective vectors (assuming progeny were virus-free) had little effect on disease incidence in most cases except where birth rates of virus-free vectors only slightly exceeded their death rate; in this case, increasing the fecundity of infective vectors increased vector population density and disease incidence.

**Figure 6:**
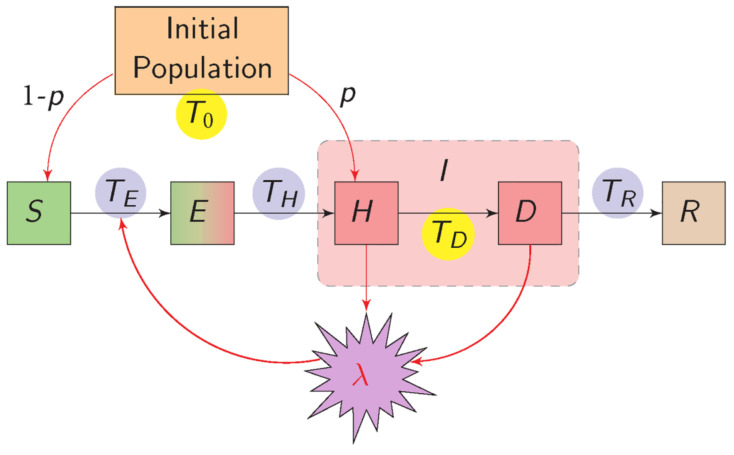
Susceptible-Exposed-Hidden-Detected-Removed (SEIR) model of disease progress in a fragmented landscape, Pleydell et al. 2018 [171], (Figure 1). The landscape consists of a set of patches across which vector dispersal allows for the spread of a virus. At time *T*_0_, a patch consists of infectious (*I*) or susceptible (*S*) host plants with probabilities *p* and 1-*p*, respectively. An individual plant moves between the compartments exposed (*E*), infectious (*H*), detected (*D*), and removed (*R*) at successive times *TE*, *TH*, *TD* and *TR*. Infection of susceptible host plants occurs at rate λ. Dispersal among patches (connectivity across all patches in the landscape) is modelled by a 2-dimensional dispersal kernel.

Dispersal, both active and passive, is a key element in the life cycle of most arthropod pests, including those that vector plant diseases [170]. It has proven difficult to monitor and estimate dispersal parameters that are relevant to disease spread observed in field epidemics. This is especially the case where disease spread is occurring over a heterogeneous and fragmented landscape. A model framework including efficient computational procedures was developed to estimate dispersal distances in such an environment and was applied to long-term and large-scale surveys of plum pox disease to estimate the flight distribution distances of infective aphids [171] (Figure 6). More than half were estimated to fly for more than 90 m, with some 10% remaining in flight for more than 1 km. This information could serve for example to inform surveillance for secondary infections arising from a primary infection in peach orchards, but the approach could be suitable for other virus diseases mediated by flying vectors.

Landing behaviour of virus vectors in crops is influenced by both visual and olfactory plant cues. Management of nonpersistent viruses has largely stressed the visual cues but knowledge of how a virus manipulates a plant to enhance its own dispersal could be used to develop traps based on olfactory cues, including border or trap crops and ‘push-pull’ systems [172].

The incidence of virus disease in a crop can depend as much on the presence of the virus and vector in alternative crops, wild plant populations and weeds, as on epidemic processes within the crop. This is particularly relevant where these alternate hosts act as a reservoir for both virus and vector and infection results from often transitory immigration and feeding of vectors into and within the crop. An epidemiological model was proposed [173] to represent such a situation for tomato leaf curl virus disease. It was found that even a low level of simulated immigration could lead to almost total infection in the crop and in most cases vector control with insecticides was only effective when used intensively and efficiently, and with low vector numbers. Combining reductions of vector immigration, for example through protective netting and infection using less susceptible varieties, was proposed as the best targeted disease management strategy for these situations.

### 5.2. Feeding Preference and Behaviour

A useful behavioural distinction can be made between colonising vs non-colonising vectors on a given crop. Based on transmission studies and spatiotemporal patterns of virus spread in melon crops, it was concluded that CMV was mainly transmitted by colonising species such as *Aphis gossypii*, whereas watermelon mosaic virus was mainly transmitted by non-colonising species such as *M. persicae* [174]. On zucchini, both *A. gossypii* and *M. persicae* are considered colonising species and were responsible for much of the epidemic spread of zucchini yellow mosaic virus compared with non-colonising species [175].

Sap-sucking insects such as aphids, whiteflies and leafhoppers while feeding on plants cause little physical tissue damage, but in feeding can acquire and transmit plant viruses. The plant’s defence response to feeding can now be studied in much greater molecular detail in real-time [176] and may be of use in determining resistance mechanisms involving acquisition and inoculation resistance, known to have an epidemiological impact without undesirable evolutionary impacts on the virus. The crinivirus cucurbit chlorotic yellows virus is transmitted by *B. tabaci* in a semi-persistent manner. Acquisition of the virus changed feeding behaviour in a biotype and sex dependent manner, but, overall, the results suggested an increased ability of the vector to transmit the virus [177]. Vector feeding behaviour can be modified to enhance the probability of transmission, as has been shown for some plant viruses belonging to the Bunyaviridae [117,118], a trait shared with animal infecting members of this virus family. The frequency of feeding of viruliferous thrips *F. occidentalis* carrying TSWV was some three-fold higher and caused less plant cell damage than with non-viruliferous thrips

Insect vectors of plant viruses are also plant herbivores. Few models have taken the dual nature of herbivory and the epidemiological consequences of infection into account. A hybrid model was developed [178] in which infection affects the nutritive quality of plants. In cases where the herbivore vector preferentially feeds on infected plants it was shown that initial conditions can in some cases allow the disease to persist even when the *R*_0_ threshold does not hold. Drought stress intensity and duration can also affect vector behaviour, feeding and reproduction. [79]. Fecundity of aphids feeding on barley yellow dwarf virus infected plants was increased by almost 50% when wheat was grown under severe water stress under glasshouse conditions; when water was not limiting, fecundity was only increased by about 20%.

For persistently transmitted viruses, the duration of the feeding period is critical in determining then probabilities of acquisition and inoculation. As pointed out [179], the variability of feeding periods is often not considered in models of transmission, but rather constant transmission coefficients are assumed, which would correspond with average feeding period durations. However, in systems in which vector transmission is relatively inefficient or low, the mean feeding period by a vector is less than that required for an acquisition or inoculation event to take place then the assumption of a constant feeding rate can lead to either an overestimate or underestimate of acquisition or inoculation.

Many virus vectors such as aphids have associations with endosymbiotic bacteria. Endosymbionts may influence aphid physiology and fitness in various ways. They may also affect feeding behaviour by increasing the number of punctures and reducing the duration of each puncture [180]. The net effect on sustained phloem ingestion and hence the probability of virus transmission may be expected to affect disease epidemiology but so far has not been studied in detail (except for their involvement in the movement of circulative viruses in vectors).

### 5.3. Tripartite and Tritrophic Interactions

Tripartite interactions occur between organisms at the same trophic level. Interactions between herbivore vectors and herbivore non-vectors have been little studied in transmission and other epidemiological studies. Experiments were conducted to determine whether defoliation by *Sitona lineatus* affected the behaviour of *Acrythosiphon pisum* the vector of pea enation mosaic virus [181]. It was found that herbivory by *S. lineatus* stimulated vector movement and virus spread to more susceptible parts of the plant. A key role of insects is to provide an ecosystem service through pollination of crops and native flora, a provision directly related to their feeding behaviour and life cycle. An intriguing suggestion made recently is that plant viruses may help to manipulate plant-insect interactions by making plants more attractive to pollinators [182].

Parasitoids and natural enemies have been shown to have a major effect on insect vector populations and behaviour. Such effects may be expected to affect virus transmission and disease spread. In a range of experimental test arenas, introduction of the parasitoid *Aphidius ervi* reduced population numbers of the aphid *A. pisum* but increased nine-fold the incidence of bean yellow mosaic virus disease [183]. These effects were interpreted as due to disturbance of the vector while feeding on an infected bean plant leading to increased movement and inoculation of new healthy plants. The evidence from experimental microcosm studies such as this was consistent with modelling studies that showed that natural enemies can, at the same time as reducing vector numbers, increase the rate of virus transmission and the rate of disease development [184]. Based on evidence from both experimental and modelling studies, a schema [185] was proposed showing how natural enemies of virus vectors can increase virus fitness by a series of signals in which: vectors are attracted to infected plants by visual or olfactory cues; a plant response attracts natural enemies to the colonised infected plant; and an alarm signal triggered by presence of the natural enemy causing dispersal of the now-infective vector to a new plant. This schema would then be complete if it could be shown that the infective vector now has preference for a healthy plant as discussed in the next section.

The consequences of tritrophic interactions for the longer-term dynamics of a plant virus disease have been less studied. The short and long-term spatiotemporal dynamics of CMV (nonpersistent) and cucurbit aphid-borne yellows virus (CABYV, persistent), both transmitted by *A. gossypii* were studied in the presence or absence of the parasitoid *Aphidius colemani* [186]. In the short term (two days), the presence of the parasitoid led to increased vector dispersal and spread of the nonpersistent virus CMV, although with benefits for disease control in the longer term. For the persistent virus CABYV, the presence of the parasitoid restricted virus spread in the longer term (14 days) largely because persistent viruses tend to be associated with longer feeding periods.

A relatively unexplored area of research concerns competition between multiple vectors in transmission of a given virus, a scenario which has been modelled in terms of the basic reproduction number [55]. In this situation, how the presence of predators/parasitoids with different prey/host preferences would affect transmission and disease dynamics is unknown.

## 6. Vector Preference

Vector preference for host plants can take many forms.

(i) A vector might show a preference for a given plant species or genotype, even though many related species/genotypes may harbour populations of the vector. This then becomes an important consideration when this range of plant species/genotypes can be shown to be susceptible (at least in artificial inoculation studies) to the virus being vectored.
(ii) Preference can also be shown for infected or healthy phenotypes of a given plant species through visual or olfactory cues.
(iii) In an elaboration of this, vectors may switch preference from infected phenotypes when nonviruliferous, to healthy phenotypes when viruliferous. This form of vector preference has been termed conditional preference and is the subject of much current research based on the premise that it represents virus manipulation of the plant and vector.

In each of these areas there have been extensive studies published—only a selected number are discussed here. Earlier papers up to about 2016 are summarised in Table 1, covering thrips [187,188,189,190,191], whiteflies [192,193,194], and aphids [195,196,197,198,199]. Other papers, representative of more recent work, are summarised in the concluding sub-section.

### 6.1. Vector-Host Range Preferences

In studies on the host range of tomato chlorosis virus, several solanaceous and unrelated plant species were found to be susceptible and which supported oviposition by *B. tabaci* [200]. In transmission studies it was found that some of the plant species served as a source of virus, although at a much lower level that infected tomato. There seemed to be no correlation with the rate of transmission from and oviposition on a given host, for example tomato as a source led to an infection rate of 76% compared with eggplant (3%). The equivalent figures for oviposition were 2.7 and 10.6 eggs/cm^2^ respectively. It seems that host preference among genotypes or cultivars of a crop may not be affected by whether a vector is viruliferous or non-viruliferous, i.e., there is no host genotype x vector status interaction. This was found for soybean and soybean vein necrosis virus where viruliferous and non-viruliferous adult female thrips were used to infest 18 soybean genotypes with the numbers of immature thrips subsequently counted [201]. There was a positive correlation between the resulting thrips counts on genotype between viruliferous and nonviruliferous thrips. However, the counts of nonviruliferous thrips were always higher than viruliferous trips in both choice and no-choice experiments. In no-choice experiments, counts of mature thrips did not differ by soybean genotype.

### 6.2. Host Phenotype Preference and Vector Performance

During the last decade a major development in plant virus epidemiology has been the recognition that vectors may show preferences for landing and feeding that are dependent on the host’s status as infected or healthy. The responsiveness of insect vectors to healthy or infected plants was described for two systems: wheat/barley yellow dwarf virus/ *Rhopalosiphum padi* and potato/potato leafroll virus/*M. persicae* [202]. Virus infection of both plants enhanced the life history of vectors; and, moreover, that the settling preferences of the vectors was mediated by volatile organic compounds. Compared with non-viruliferous vectors, viruliferous vectors were less or not responsive to these compounds but this did not at the time indicate a switch in preference to healthy plants. Host phenotype preference in relation to host resistance/tolerance can be more complex with interactive effects of vector preference and host defence [203]. Complex effects of co-infection compared with single infection both on within-plant chemistry and host-vector interaction were described for BPMV and soybean mosaic virus [204].

**Figure 7:**
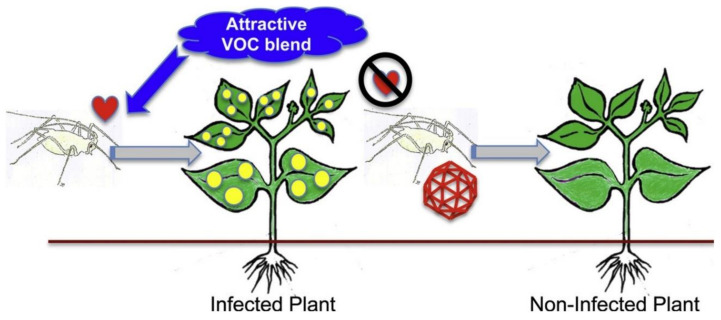
Schematic representation of the attract and deter host plant phenotype, Carr et al. 2020 [205], (Figure 1). In some non-persistently transmitted viruses, an infected plant gives off Volatile Organic Compounds which attract the vector (in this representation an aphid) to land and probe epidermal cells. However, virus infection may result in plant chemicals which deter the vector from settling and feeding, with the vector now potentially having acquired virus from the initial probing moving on to potentially inoculate a healthy plant.

For non-persistent transmission, it has been argued that inhibition of settling while allowing probing will encourage transmission, whereas prolonged settling will retard transmission (Figure 7). However, recent mathematical modelling indicates that both virus-induced effects will contribute to epidemic development at different scales [205]. This is because the “attract and deter” virally modified host phenotype will favour local and short-term spread of virus, whereas a virus induced “retain” host phenotype will favour longer-term and larger-scale spread, in cases where vector crowding leads the production of alate forms which carry virus over longer distances.

As well as the responses of insect vectors to virus infections, effects may also be found with non-vector insect herbivores and interactions with predators, as discussed in the section on tripartite and tritrophic interactions. CMV-infected plants may well have a reduced overall herbivore pressure through effects on palatability and apparency, however predators were found to locate herbivorous prey on infected plants as efficiently as those on healthy plants [206].

### 6.3. Conditional Vector Preference and Virus Manipulation

Just as plant viruses can affect insect vector behaviour and population dynamics as described in the previous section [6], these aspects of the vector can affect the transmission of viruses and disease dynamics. The question remains as to whether the virus is manipulating the plant and indeed the vector to increase fitness. Experimental evidence for changes in vector behaviour induced by host infection status was made quite early [207]. Persistent and nonpersistent viruses will have different effects on vector preferences for alighting, settling and dispersal on infected and healthy plants [208]. It seems that any difference in effect found depends on whether settling or probing was evaluated [209,210]. The evidence that the molecular mechanisms for determining vector preference are associated with virus components supports the view that viruses manipulate both host and vector to enhance transmission [211].

There is now increasing acceptance that plant viruses have adaptations that facilitate transmission through acquisition, retention and inoculation. There appears to be a remarkable degree of convergence among unrelated viruses that share similar transmission characteristics, although the specific details of the virus-vector-plant interaction can clearly differ [211]. Importantly this review tests some key predictions based on whether there is nonpersistent, semi-persistent or circulative-persistent transmission, with adaptive manipulation most apparent in the latter case. However, as also pointed out [212] although the main factors influencing transmission and selection for manipulative traits have been identified, there are important gaps in linking findings with evolutionary processes in the field.

Mathematical modelling has also contributed to analysis of the epidemiological consequences of vector preferences. The landing and feeding preference of insect vectors on infected or healthy host plants has been shown to depend on whether the vector is inoculative. Including conditional preference depending on vector status into models, showed that a switch in preference once vector acquire virus from infected plants can enhance spread throughout an epidemic in cases where non-inoculative vectors prefer infected plants and inoculative vectors prefer healthy plants [213]. This important result expands on previous modelling work which suggested that vector preference for diseased plants was advantageous (for the virus) when disease incidence was low, and vector preference for healthy plants was advantageous when disease incidence was high. A more comprehensive model with conditional vector preferences but also including more vector life history traits has been developed [214]. Traits including intrinsic growth rate and population carrying capacity, as well as landing and departure rates, were introduced conditional upon whether the host is healthy or infected and whether the vector is viruliferous or nonviruliferous. The key result from numerous simulations based on parameter values for barley yellow dwarf virus and PVY indicated that vector population growth rates overall had the greatest effect on virus spread, but also that rates of vector dispersal from infected hosts and from hosts of the same virus status as the vector were also important. These interpretations were based mainly on numerical simulations of the model. A simpler approach to analysing the consequences of vector preference in a mathematical model would be to derive the basic reproduction number, as outlined in the Appendix A.

### 6.4. Other Recent Work

Since the published studies listed in Table 1, further insight into the epidemiological consequences of vector preferences has been obtained. Links with vector-natural enemy associations have been confirmed [215]. Tomato infected with tomato yellow leaf curl virus changed the host preference of the parasitoid *Encarsia formosa* between Q- and B-biotypes of *B. tabaci*: on infected but not healthy plants the Q-biotype was more attractive to the parasitoid than the B-biotype, due to quantitative differences in volatile profiles. Thrips species have long been reported to have feeding preferences for tospovirus infected plants. Virus acquisition occurs during the developmental period from early instars to adults and this period was shortened when *Thrips palmi* had acquired virus from groundnut bud necrosis virus infected plants without any effect on pre-adult mortality [216]. Squash vein yellowing virus infects both squash and other cucurbits including watermelon. More *B. tabaci* whiteflies landed and settled on infected than non-infected squash, but the opposite was found with watermelon [217]. In addition, whiteflies laid many more eggs on non-infected watermelon, but no differences were observed on squash. The time from egg laying to adult emergence was shorter, females lived longer and were more fecund on infected squash. Whitefly behaviour differed between the two cucurbit hosts but integrating the various life history traits into a comparison of disease dynamics in the field problematic.

It should also be noted that vector life history trait preferences are not always consistent across different hosts, even when virus-infected plants are generally higher quality hosts [218]. The effect of insects feeding on plants infected with viruses they do not vector has also been studied. When *B. tabaci* preferentially feeds on different hosts infected with TSWV, body size, longevity and fecundity were all reduced [219] indicating that the initial preference for a virus-infected plant was induced by host volatiles and not by subsequent performance. Host volatiles are not always the cue for vector preference. With the beetle-transmitted BPMV, the beetles are more attracted to infected soybean and it was also found by modulating sucrose levels across near-isogenic lines of soybean that although beetles consumed less leaf foliage per plant, they fed on more plants per unit of time if they had high levels of sucrose [220]. Pea aphid clones adapted to either pea or alfalfa were tested to see how bean leafroll virus affects their performance and preference [221]. Aphid clone x host plant species x virus status interactions and unique virus-association phenotypes were found, with consequences for host plant use and disease epidemiology. The virus-induced changes in host phenotype that affect vector dispersal and disease spread depend on several factors related to transmission mode and efficiency [222]. The brassica species *Camelina sativa* was infected with the cauliflower mosaic virus (CaMV, non-circulative) or turnip yellows virus (TuYV, circulative) with the aphid vectors *M. persicae* (generalist) and *Brevicoryne brassicae* (specialist), differing in transmission efficiency. Results showed that negative host-mediated effects of the nonpersistent CaMV on feeding behaviour and performance of both aphid species enhanced virus fitness. Transmission efficiency also played a role as only the response of the specialist vector to host-mediated effects of TuYV played a role in increased dispersal.

### 6.5. Future Opportunities

A key question in future research is does a link between vector preference, transmission type, and natural enemies lead to increased virus fitness and can this be represented in the basic reproduction number? It is difficult to see how hypotheses on epidemic dynamics given different forms of vector preference could be tested in the field, which returns to the point made in the introduction about the balance that needs to be struck between explanation and prediction. This is one reason why modelling can assist in creating and analysing different scenarios. Modelling, for example, can help to differentiate the consequences of non-conditional and conditional preferences, even though these are unlikely to be tested in the field.

## 7. Co-Infection

Co-infection by multiple virus species or strains is known to be common, but presents major problems in etiological and epidemiological studies, not least where detection and identification are required for the causal agents. Improved diagnostic procedures, such as high throughput sequencing, introduce new problems in determining pathogenicity, assigning causality to a single agent in the mixture or to interactions among the co-infecting entities, and identifying novel or cryptic viruses [223].

The literature on co-infection by plant viruses is extensive. In September 2017, a literature search yielded some 180 papers a mixture of reviews, perspectives and original research. Most papers were concerned with interactions between viruses at different levels dealing with cellular (even nuclear) interactions, cell-to-cell movement, vector transmission, virulence, symptom development, and yield losses. Cellular interactions were the most prevalent, mostly replication rates and virus titre (not necessarily correlated), but some studies showed clear interactions with vectors over short (epidemiological) and long-term (evolutionary) time scales. Some of the publications reflect some aspect of co-infection in relation to vector transmission, but often the vector contribution to coinfection is not discussed. Coinfections can also arise from vertical transmission [224]. Even when a plant disease has been long-known and studied, such as sugarcane mosaic virus [225], evaluation of its damaging effects can be made difficult by the widespread occurrence of coinfection with other viruses.

### 7.1. Methodological Issues

There are key questions over what is meant by “simultaneous” and “synergy/antagonism” in relation to virus co-infection which modelling and statistical analysis could bring some clarity and rigour.

Syller [226] reviewed the literature on “simultaneous” transmission of plant viruses by vectors. Most studies have been done with non-persistent transmission by aphids and emphasise the acquisition component of transmission with little consideration of inoculation. The concept of “simultaneous” is not made entirely clear, whether it can only occur at the overlapping region where there is spatial separation between two viruses and acquisition can occur during a single probe. Also, the review recognises that, for non-persistent transmission, with a following probe on the same or different plant one of the viruses can be detached and no longer be available for inoculation. For inoculation the question of co-inoculation becomes problematic, even more so where the vector has acquired viruses with differing transmission parameters, such as nonpersistent and persistent.

In a subsequent review, Syller and Grupa [227] differentiate between simultaneous inoculation (which they refer to as co-infection) and sequential inoculation (which they call super-infection). However, it would be difficult in the extreme to differentiate between these two outcomes, given conventional inoculation access period assays. Firstly, simultaneous is not synonymous with instantaneous. Secondly, how much time must elapse from an inoculation with a single virus before inoculation with a second virus can be considered sequential? They claim that within-plant synergistic interactions most often arise between unrelated viruses. Synergism is defined as a facilitative effect in which accumulation of one or both viruses in the host plant increases, in the case of just one virus often called asymmetric synergism by others [228]. Synergy is also used to describe more severe disease symptoms than induced by either virus alone. No formal criteria are given which specify a quantitative threshold or other condition which would justify the use of the term synergy. The review concentrates more on the molecular basis of antagonism, such as cross protection [228] or, as has been termed, super-infection exclusion. The review by Mascia and Gallitelli [229] notes the contribution that mathematical modelling could make on synergy and antagonism.

Outcomes of interactions among co-infecting entities can be neutral, antagonistic or synergistic and present statistical challenges in distinguishing these outcomes. In other areas, such as interactions between pesticides or microbial biocontrol agents in mixtures [230], rigorous statistical procedures have been developed. The simplest formula that has been used is Abbott’s formula which is equivalent to an expected outcome based on independent action but with overlap (Bliss independence), so in general the expected outcome of two chemical or microbial pesticides in mixtures would be less than their additive effect (*E*_m_ = *E*_1_ + *E*_2_ − *E*_1_*E*_2_, where the *E*’s represent proportionate effects). An outcome less than the expectation *E*_m_ would represent antagonism, an outcome greater than the outcome would represent synergy, even though it may be less than additive. The Abbott formula has been proposed as one way to study the outcome of interactions with co-infecting viruses [231], where the expected outcome, or proportionate effect, is based on disease severity and/or growth reduction in single and mixed infections of CMV and bean yellow mosaic virus in *Phaseolus vulgaris* and *Vicia fabae*. Hence, Abbott’s formula would give a threshold to determine whether there is synergy (or antagonism) between two coinfecting viruses and could be modified for effects such as on virus titre *in planta*. The claim of synergy or antagonism among co-infecting viruses or virus strains is now so pervasive that rigorous statistical standards need to be developed to validate these claims.

In this section, co-infection will be dealt with in stages from two viruses—with a common vector to many viruses—with many vectors, but with some nuances. Many of the papers acknowledge that there are several or many (in the case of aphids) vectors of a given plant virus but the experiments reported in general only involve one vector. Similarly, the same virus and vector can infect more than one crop (CMV is an extreme example) and hence cause more than one disease. These are perennial problems in the plant virology literature. Questions whether the helper strategy, in which a helper virus (normally avirulent) can be transmitted by the vector but the dependent virus (normally the virulent one) can only be transmitted in the presence of the helper [232], falls within the scope of the review.

### 7.2. One Vector Species

A recombinant necrotic strain of PVY (PVY^NTN^) has increased in the US (and elsewhere) while the wild-type strain PVY^O^ has been decreasing [233]. Transmission efficiency was determined in experiments where virus was acquired sequentially by an individual aphid (*M. persicae*) or by separate aphids and then inoculated to the same plant. The necrotic strain was transmitted more efficiently than the wild type irrespective of the order of acquisition or inoculation. Rarely was it found that aphids sequentially acquiring both strains co-inoculated a recipient plant. An aphid does not to acquire both strains from one co-infected plant in order to transmit both strains. Co-infected plants would more likely result from inoculation by multiple aphids feeding on plants infected with the different strains rather than by single aphids feeding on multiple plants infected with the different strains.

Previous work had suggested some specificity in transmission of strains with rate of infection for PVY^NTN^ higher than for other strains, a vector-related outcome as the same outcome was not observed with mechanical transmission [234]. A more complex set of experiments was reported by Mondal et al. [235] involving three PVY strains (two isolates of each strain) and three aphid species as vectors. According to Syller [226], within-plant antagonism occurs most often with related strains, but does this translate across to effects on transmission and epidemic dynamics?

Two PVY strains, one virulent and one avirulent, were transmitted to *Capsicum annuum* by *M. persicae* using standard transmission procedures [140]. Both were equally transmissible and competition between them was studied to estimate the size of bottlenecks imposed by vector transmission. If there was a cost of virulence, simulation modelling showed that virulent strains would go extinct and the model estimated the number of transmission events necessary for this to occur.

Oats were inoculated with two virus species barley yellow dwarf virus (BYDV- PAV) and cereal yellow dwarf virus (CYDV-RPV) with the aphid *R. padi* [236]. The co-inoculation of BYDV-PAV lowered CYDV-RPV infection rate, but this occurred at low nutrient (N/P) supply and was not present at high supply rates. This provides a useful reminder that broader environmental and nutritional factors can alter co-infection interactions and outcomes.

Single and mixed infections were studied for two potyviruses, watermelon mosaic virus (WMV) and zucchini yellow mosaic virus (ZYMV), commonly infecting cucurbitaceous crops [237]. The study crop was squash and the aphid vector *A. gossypii*. ZYMV accumulated at similar rates in single and mixed infections, whereas WMV was much reduced in the presence of ZYMV. ZYMV also induced host changes that gave strong vector preference for infected plants, whereas WMV did not, although it was still readily acquired from mixed infections.

### 7.3. Two Vector Species

BPMV and soybean mosaic virus (SMV) both cause substantial yield losses in soybean, losses which are worsened by co-infection with little or no harvestable yield. BPMV is transmitted by beetles including the coccinelid *Epilachna varivestis*; SMV by aphids including the recently introduced *Aphis glycines*. Penaflor et al. [204] studied the acquisition and inoculation of each virus and its vector in single infections, the likelihood of secondary infection for singly infected plants, and whether co-infection affects transmission for each virus. Singly infected plants with either BPMV or SMV increased soybean palatability, potentially enhancing acquisition of BPMV from BPMV plants and secondary infection of BPMV from SMV plants. Co-infected plants were not more palatable to beetles. BPMV had little effect on *A. glycines*, whereas SMV infection reduced aphid population growth but conversely increased the preference for infected plants. Coinfection reversed the effects on population growth and aphids showed a preference for co-infected plants.

Torradoviruses are spherical viruses transmitted by the whitefly vectors *Trialeurodes vaporariorom* and *B. tabaci*. The group is of interest because it does not fall neatly into the transmissions categories of stylet-borne or semi-persistent (foregut). At least three whitefly species have been shown to transmit four torradovirus species [126] but the experimental details do not include co-infected plants.

Complications in analysing co-infection data can arise when two distinct viruses are transmitted by two distinct species of vector. Such as situation arises with data on Citrus leprosis virus C and orchis fleck dichorhaviris citrus strain [238] incidence in field populations of the mite vectors *Brevipalpus yothersi* and *B. californicus*, collected in citrus orchards. The rounded incidences were 17, 10 and 3% for singly and co-infected vectors respectively in *B. yothersi* and 12%, 21% and 4% in *B. californicum*. Clearly the incidences of coinfection in the two mite species were greater than the product of the two single incidences [239].

### 7.4. Many Vector Species

Two viruses are responsible for RTD: rice tungro spherical virus (RTSV) and rice tungro bacilliform virus (RTBV). They are both transmitted by leafhoppers in a semi-persistent manner with the most efficient vector being *Nephottetix virescens*. Infection by each virus alone results in less pronounced symptoms especially for RTSV. RTBV is retained in the vector for a longer period, 4 rather than two days. When a vector carries both viruses, co-inoculation seems to be common. When the vector is inoculative with RSTV alone the infection probability seems to be higher. A simulation model with assumptions based on these and other details was developed by Holt and Chancellor [96] to examine whether spread could be prevented by rogueing of diseased plants. It was found that, with medium-to-high simulated disease levels, rogueing was ineffective even when applied efficiently; with low simulated disease levels, rogueing was effective but of little practical value.

Grapevine leafroll disease is caused by a range of monopartite closteroviruses designated as Grapevine leafroll-associated viruses (GLRaVs), although their exact role in disease etiology remains unclear [240]. The transmission of GLRaVs is through mealybugs and scale insects. With mealybugs transmission is of a semi-persistent manner with a lack of vector-virus specificity. Co-infections of GLRaVs are frequent in grapevines although with some spatial separation with implications for transmission and epidemiology

SPVD results from co-infection with sweet potato chlorotic stunt virus and sweet potato feathery mottle virus. At least six other viruses from the same or different virus families were found to interact synergistically with SPVD [241]. The claimed synergism was based on the increase in disease symptoms, virus accumulation and movement in plants, and reduced yield of storage roots. No criterion was presented for synergistic interaction and as no vectors were involved (all inoculations were made by grafting) no conclusions can be drawn on vector transmission effects.

In field studies of barley and cereal yellow dwarf viruses in US west coast grasslands, the effect of shared vectors, environmental conditions and spatial variation in hosts affected the spatial variation of single viruses, paired viruses and the whole virus community in grass hosts [242]. It was found that for single viruses and the whole virus community, there was a random spatial distribution which was considered to reflect a random pattern of spread. By contrast, for paired viruses which shared a common vector there was an aggregated spatial distribution, interpreted as due to environmental conditions, spatial variation in hosts and vector preferences.

At a high level, the ecological networks formed by multiple co-infecting viruses, hosts and vectors were analysed by McLeish et al. [243]. Co-infection networks were found to lead to strong non-random associations compared with single infections. Single infections were mostly related to habitat parameters, whereas co-infections were more related to ecological heterogeneity and ecosystem-level processes. Network analysis has been applied within and between hierarchical levels as shown recently in analysis of the papaya orchard virome in two contrasting agro-ecological zones in Mexico [244]. An introduction to the use of network analysis in plant disease epidemiology was given by Jeger et al. [245] and elaborated further at scales ranging from the molecular to the landscape and global levels [246].

### 7.5. Co-Infection and Vector Preference

The previous section examined how vector preference can affect the transmission and disease spread of plant viruses. In this section the epidemiology of co-infecting plant viruses is being examined. An important consideration in this context is the extent to which co-infected plants affect vector preference compared with plants infected with a single virus. Aphids (*Amphorophora agathonica*) preferred to land on raspberry leaf mottle infected plants rather than healthy plants, but on healthy rather than raspberry latent virus infected plants [247]. No settling preferences for co-infected compared with healthy plants. It was found that both non-viruliferous and viruliferous whiteflies with the begomovirus cucurbit leaf crumple virus and/or the crinivirus cucurbit yellow stunting disorder virus preferred non-infected plants over infected plants. It appeared that whitefly preference was not affected following individual or combined virus acquisition [248].

A novel approach was taken in which virus effects on host-vector interactions affected competition between co-infecting viruses [237]. Watermelon mosaic virus and zucchini yellow mosaic virus share the same aphid vector and frequently occur in mixed infection, with ZYMV multiplying more than WMV in mixed infections compared with their multiplication in single infections. In addition, ZYMV induced host visual and olfactory cues that increased *A. gossypii* landings on infected plants, whereas WMV did not. Despite these apparent disadvantages, WMV was readily transmitted from co-infected plants despite performing poorly within plants. Thus, the increased transmission arising from ZYMV in mixed infections benefitted the less competitive WMV.

Another example where vector preferences has been found to increase levels of co-infection is found with southern rice black-streaked dwarf virus (SRBSDV) and rice ragged stunt virus (RRSV) [249]. Both viruses are transmitted by the planthoppers white-backed planthopper (WBPH) and brown planthopper (BPH) in a persistent-propagative manner. Non-viruliferous WBPH significantly preferred infected rice plants whereas both viruliferous and non-viruliferous preferred healthy plants. Non-viruliferous BPH preferred healthy plants whereas BPH carrying RRSV preferred SRBSDV-infected plants, thus enhancing spread and resulting co-infection.

### 7.6. Co-Infection and Segmented Viruses

**Figure 8:**
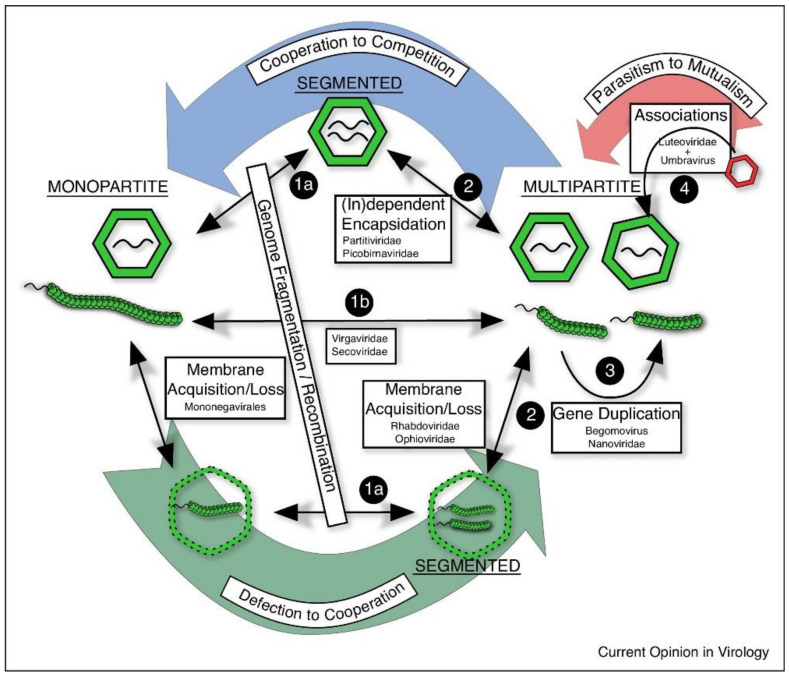
Possible evolutionary pathways to multipartite virus form from a monopartite ancestor [256], (Figure 1). The epidemiological contribution to the main pathways, involving cooperation, competition, parasitism and mutualism, are not represented in this schematic.

Cucumber mosaic is a tripartite RNA virus with the genomic RNA packaged into separate particles [22]. Multiple virus particles must enter a plant cell (more than 1000 known host plant species) to initiate an infection, requiring a multiplicity of inoculation events by aphid vectors (more than 80 species known to transmit). Reassortment mechanisms may have contributed to the genetic diversity found and the evolutionary success of the virus. Many of the begomoviruses have bipartite genomes (DNA-A and DNA-B), but the role of the complete genome in whitefly transmission has been little studied. An increasing number of papers are appearing on multipartite viruses, especially how these may have arisen from monopartite viruses (Figure 8), where each segment is separately encapsidated and all are necessary for a fully functional virus in the plant [250,251,252,253,254,255,256]. As stated in the title “keep connected or die” [257], the multipartite life strategy raises epidemiological questions, especially concerning transmission and the vector–virus relationship, and about how such viruses can persist in a host population.

### 7.7. Future Opportunities

The area of co-infection and how this relates to recent work on vector behaviour and preference and at a higher scale the agroecological consequences is ripe for further attention by mathematical modellers [258,259,260,261,262]. More generally, the key new element in plant virus epidemiology is how to deal with co-infection with different vectors, transmission types, vector preferences, and other non-viral pathogens, how these then translate into ecological and evolutionary consequences and vice-versa, and how can control strategies best be devised.

## 8. Concluding Comments

There has been considerable progress over the last decade in achieving a greater synthesis of ecological and evolutionary insights with plant virus diseases and how these affect crops and wild plant communities. The role and function of plant viruses in nature have received a greater appreciation, for example the evolutionary trajectories that are possible from pathogenicity to mutualism, and vice-versa [31]. However further evidence is required that quantitative epidemiological analysis can help to describe and explain short-term evolution and can be used in disease management [32]. Equally, the ecology of domesticated crops and farming systems and their impact on plant viruses and pathogens more generally needs as much attention as has been given to wild plant communities [38,40,41].

Methods of epidemiological analysis have also matured over the last decade from the simple description of disease progress curves and disease gradients. Modern computational and data mining techniques have transformed the ways in which theoretical epidemiological models can be evaluated and parameters estimated when extensive empirical data on disease progress and associated biotic and abiotic factors are available, e.g., the linking of Markov chain Monte Carlo (MCMC) methods to estimate the parameters of compartmental models of disease progress [49,50]. There needs to be a greater attempt to calculate the basic reproduction number from field data [52] rather than deriving an appropriate expression from theoretical models.

Considerably more work has been done on characterising spatial aggregation/structure in landscapes as a basis for describing and in some cases predicting the consequences of a plant virus epidemic [70,71,72]. Compared with fungal plant pathogens, there is a need for further evidence on climate change effects on plant viruses in crops and wild populations [75] especially where there are different effects on host growth and aphid performance depending on virus infection [263]. In terms of linking epidemiological research and analysis to disease management, there is now a greater recognition that appropriate spatial and temporal scales are required for forecasting [82,83]. Crop heterogeneity is known to mitigate the population build-up of arthropod pests and the damage they cause, and these effects should be extended to consequent effects on vectored plant virus epidemics [103,105,107]. In general, despite the general acceptance of Integrated Pest Management as being beneficial in arthropod pest control, it has not been fully integrated into devising and implementing integrated disease control measures for plant viruses [109,110,111,112]. A promising approach is to build on earlier empirical studies for predicting vector-related aspects of virus epidemics, such as Plumb’s infectivity index for BYDV, by process-driven models [264].

Perhaps the most promising development over the last decade has been the greater recognition of, and prominence given to, the vector in all aspects of virus epidemiology, and indeed this is a common thread from the ecological and evolutionary perspectives as well. How best to deploy host resistance and tolerance in the field can only be determined if the dynamic interplay between the virus, vector and host is characterised. The consequences of releasing varieties with different mechanisms of resistance can differ and affect the effective use of a variety [265,266,267]. The key role of vector transmission in virus epidemiology has moved on from simple classification to deeper insights; for example, how the interactions between horizontal and vertical transmission can have significant epidemiologically and evolutionary consequences [268,269]. Much of this insight has been gained from theoretical models and limited laboratory/microcosm experiments and a challenge for the future will be to scale up to the field. A striking feature that exemplifies this need is the work that has been done on vector preference and the extent to which the virus is manipulating the vector and the host to its own advantage. Important work has been done on determining the molecular basis for vector preference [270], but the challenge is to translate this to the epidemiological and evolutionary consequences.

Co-infection with plant viruses is now recognised as the norm for many crops. However, to what extent is such co-infection significant or simply a consequence of recent developments in high throughput sequencing [223]. There are clearly examples in which co-infection can lead to greater damage in a crop than do single viruses, but there needs to be more attention to how two or more viruses are interacting within the plant and much greater rigour in defining the outcome of such interactions, whether antagonistic, neutral or facilitative (use of the term synergistic demands considerable caution). Even when these interactions are characterised, there is still the need for more work on how these are expressed in an epidemic [239,262]. There have been studies on how coinfection may or may not affect vector preference, but generally the role of the vector in maintaining coinfection in a plant population has been under-explored. Finally, although multipartite viruses are not normally considered under the umbrella of coinfection, for those which have genomic segments that are encapsidated separately and may require separate inoculations by a vector for a fully functional virus to be present in a plant cell, the question remains: how do multipartite viruses even persist in a plant population [256]?

## Figures and Tables

**Table 1:** A summary of selected publications on vector preference in the period to 2016. Key: A—Preference of non-viruliferous vectors for infected plants, B—Preference of viruliferous vectors for healthy plants.

Virus	Host	Vector	A	B	Additional Comments	Ref.
Soybean vein necrosis virus	Soybean	*Neohydatothrips variabilis*	?	Y	Viruliferous vectors produced more offspring but excessive accumulation led to lower viability	[187]
*Tomato spotted wilt virus*	Pepper	*Frankliniella occidentalis*	Y	Y	Exposure to TSWV as larvae gave shorter developmental times	[188]
*Tomato spotted wilt virus*	Arabidpsis thaliana	*Frankliniella occidentalis*/*Thrips tabaci*	?	?	Plants infected with a non-transmissible thrips strain were preferred over uninfected plants. Transmissibility by thrips of TSWV was unrelated to vector preference	[189]
Watermelon silver mottle virus (P)	Watermelon	*Thrips palmi*	Y	N	T palmi also preferred feeding on thrips -damaged plants to healthy plants. Mixed effect on thrips performance parameters	[190]
*Tomato spotted wilt virus* (P)	Peanut	*Frankliniella fusca*	?	?	Preference refers to “speed of feeding” of non-viruliferous compared with viruliferous F fusca	[191]
Tomato chlorosis virus (SP)/Tomato severe rugose virus (P)	Tomato	*Bemisia tabaci*	?	?	ToSRV whiteflies preferred volatiles from non-infected plants; non-viruliferous whiteflies avoided volatiles from ToCV infected plants	[192]
Tomato yellow leaf curl virus (P)	Tomato	*Bemisia tabaci*	Y	Y	Preferences were only prominent on a susceptible rather than resistant genotype. Developmental time was only reduced on TYLCV-infected plants	[193]
Tomato yellow leaf curl virus (P)	Tomato	*Bemisia tabaci*	Y/N	N	Virus-free Q-type preferred TYLCV infected plants; virus-free B-type preferred healthy plants. TYLCV whiteflies (both Q and B) show no preference for TYLCV-infected or virus-free plants	[194]
Barley yellow dwarf virus	Wheat	*Rhopalosiphum padi*	Y	N	Non-viruliferous preference was not affected by plants co-infected with *Gibberella zeae*, which also supported greater population growth	[195]
Cardamon bushy dwarf virus	Cardamon	*Micromyzus kalimpongensis*	?	Y	Aphids grown on CBDV plants had shortened nymphal periods and increased longevity and fecundity	[196]
Pea enation mosaic virus (P)/Bean leaf roll virus (P)	Pea	*Acyrthrosiphum pisum*	Y	?	The two viruses differed in their distribution within the plant, but aphids did not discriminate between plants infected by the two viruses. There was earlier nymph production on both infected plants but divergent age specific effects depending on the virus	[197]
*Cucumber mosaic virus* (NP)	Squash/Pepper	*Aphis gossypii*	Y	?	Isolates from squash induced in squash the type of preference behaviour previously found. An isolate from pepper on pepper was more neutral. Cross-host inoculations showed (mal)adaptive effects.	[198]
Sweet potato potyviruses	Sweet potato and Ipomea weeds	*Myzus persicae*	Y/N	?	In sweet potato there was preference for virus-infected plants. In the Ipomea weeds, there was preference for noninfected plants	[199]

## Appendix A. Basic Reproductive Number with Conditional Vector Preference

Vector preferences can be represented by a term which represents the bias for a vector to land on an infected or a healthy plant, as done in a model for mosquito preference for healthy or infected humans [271]. Introducing conditional preferences, the probabilities that a non-viruliferous vector lands on an healthy plant can be written as $\frac{S}{S+v_{X}I}$, and on an infected plant as $\frac{v_{X}I}{S+v_{X}I}$, where $v_{X}$ is the degree to which a nonviruliferous vector prefers to land on an infected plant: for $v_{X}$ > 1, the bias is for infected hosts; for $v_{X}$ < 1, the bias is for healthy plants. Similarly, the probabilities that a viruliferous vector will land on a healthy plant can be written as $\frac{S}{S+v_{Z}I}$, and on an infected plant as $\frac{v_{Z}I}{S+v_{Z}I}$, where $v_{Z}$ is the degree to which a viruliferous vector prefers to land on a healthy plant: for $v_{Z}$ > 1, the bias is for healthy hosts; for $v_{Z}$ < 1, the bias is for infected hosts. In both cases, the probabilities add to 1, as required.

An unpublished model (Cunniffe, N. Epidemiological consequences of virus infection altering vector behaviour. Cambridge, 2014), unlike previous models [137,138] was formulated to track disease progress with and without population dynamics of the vector and host plant. The aim was to explore in detail differences in landing, probing and feeding behaviour arising from conditional vector preference when transmission was nonpersistent or persistent, and to derive an expression for the initial rate of disease increase. based on four linked differential equations was then developed in which acquisition (with probability α) and inoculation (with probability b), are modified by these preference terms, together with the number of visits per nonviruliferous ($\phi_{X}$) and viruliferous ($\phi_{Z}$) vectors per unit time. Terms were also specified for: the proportion of visits that lead to acquisition (χ) and inoculation (ψ); plant mortality, both natural (ρ) and disease-induced (μ); and vector mortality (α) and loss of virus (τ). In the absence of the virus, the host population size was N and the vector population size was κ.

A full version of this model is in preparation. In this Appendix, a simplified form of the model is used to show how a derived basic reproduction number can provide qualitative insight into the consequences of assumptions concerning vector preferences. The labelling of $v_{X}$ and $v_{Z}$ is retained but it is assumed that $\phi =\phi_{X}=\phi_{Z}$, i.e., carrying the virus does not affect the in-flight behaviour of the vector.

A general basic reproduction number $R_{0}^{2}$ is then derived as:

(A1) $$R_{0}^{2}=\;\kappa \frac{1}{\rho +\mu }\psi \phi a\frac{v_{X}}{N+v_{X}}\frac{1}{\tau +\alpha }\chi \phi b\frac{N}{N+v_{Z}}$$

The squared formulation reflects the fact that there are two cycles involved in transmission, from plant to vector and from vector to plant. Clearly, $R_{0}^{2}$ ≥ 0 for non-negative values of the parameters. When $v_{X}$ = 0, $R_{0}^{2}$ = 0; and as $v_{Z}$ becomes large, $R_{0}^{2}$ also approaches 0. Consider a path in the parameter space (pairwise combinations of $v_{X}$ and $v_{Z}$ as both move away from the origin) with other parameters fixed. It can be shown that $R_{0}^{2}$, the number of secondary infected plants arising from one infectious plant, will be at a maximum at some point along this path.

In the baseline case where there is no vector bias, $v=\;v_{X\;}=\;v_{Z}\;=$ 1. For large *N*, $\frac{N}{N+1}$ ~ 1 and $\frac{1}{N+1}$ ~ $\frac{1}{N}$, so substituting these terms in Equation (A1) and re-arranging, the $R_{0}^{2}$ expression can be written as:

(A2) $$R_{0}^{2}\left(v=1\right)=\frac{\kappa }{N}\;\frac{1}{\rho +\mu }\phi \psi a\frac{1}{\tau +\alpha }\phi \chi b\;$$

Note that $\frac{\kappa }{N}$ is now the number of vectors per plant. The successive terms in this expression can be interpreted as follows:

Number of vectors per plant in absence of virus × average infectious period of a single infected plant × number of visits per non-viruliferous vector per day × proportion of visits that could lead to acquisition × probability of virus acquisition by a single vector × average period a vector remains viruliferous × number of visits per viruliferous vector per day × proportion of visits that could lead to inoculation × probability of inoculation by a single viruliferous vector.

Consider now the cases where vector preferences are asymmetric. In the case where vector bias applies only to non-viruliferous vectors preferring to land on an infected plant, with no such preference for a viruliferous vector to land on a healthy plant, then $v_{X}>1,\;v_{Z}$ = 1. Substituting in Equation (A1) and re-arranging so that $\frac{\kappa }{N}$ is again the number of vectors per plant

$$R_{0}^{2}\left(v_{Z}=1\right)=\frac{\kappa }{N}\;\frac{1}{\rho +\mu }\phi \psi a\frac{1}{\tau +\alpha }\phi \chi b\frac{v_{X}}{N+v_{X}}\;N\;\;\;=R_{0}^{2}\left(v=1\right)\times \;\frac{v_{X}}{N+v_{X}}N$$

As $v_{X}>1$, the multiplier is greater than 1 and the basic reproductive number will be greater than the baseline case. As $v_{X}$, the strength of preference for infected plants by non-viruliferous vectors, becomes large, the multiplier increases monotonically with the host population size. Hence, in cases where $R_{0}^{2}\left(v=1\right)<1$, for some value of N the new basic reproduction number $R_{0}^{2}\left(v_{Z}=1\right)>1$. So, a preference for non-viruliferous vectors to land on a diseased plant will be advantageous (for the virus) even in the case where there is no preference for a viruliferous vector to land on a healthy plant.

Similarly, in the case where vector bias only applies to viruliferous vectors preferring to land on a healthy plant, with no such preference for a non-viruliferous vector to land on an infected plant, then $v_{Z}>1,\;v_{X}$ = 1. Substituting in Equation (A1) and re-arranging so that $\frac{\kappa }{N}$ is again the number of vectors per plant

$$R_{0}^{2}\left(v_{X\;}=1\right)=\frac{\kappa }{N}\;\frac{1}{\rho +\mu }\phi \psi a\frac{1}{\tau +\alpha }\phi \chi b\frac{N}{N+v_{Z}}\;=R_{0}^{2}\left(v=1\right)\times \;\frac{N}{N+v_{Z}}\;$$

As $v_{Z}>1,$ the multiplier is less than 1 and the basic reproduction number will be less than the baseline case. As $v_{Z}$, the strength of preference for healthy plants by viruliferous vectors, becomes large, the multiplier decreases monotonically to approach 0. Hence in cases where $R_{0}^{2}\left(v=1\right)>1$, for some value of $v_{Z}$ the new basic reproduction number $R_{0}^{2}\left(v_{X}=1\right)<1$. So, if there is no preference for a non-viruliferous vector to land on a diseased plant, there is no advantage (for the virus) for a viruliferous vector preferring to land on a healthy plant.

Finally, in the case where vector preferences are symmetric, the biases can be: $v_{X}$, $v_{Z}$ > 1, or $v_{X}$, $v_{Z}$ < 1. For these special cases, Equation (A1) can be used to determine the value of $R_{0}^{2}$, and how this compares with the baseline $R_{0}^{2}\left(v=1\right)$, where there are no vector preferences.
